# ﻿Potential predictive value of phylogenetic novelties in clinical fungi, illustrated by *Histoplasma*

**DOI:** 10.3897/imafungus.16.145658

**Published:** 2025-05-23

**Authors:** Yu Quan, Xin Zhou, Ricardo Belmonte-Lopes, Na Li, Retno Wahyuningsih, Anuradha Chowdhary, David L. Hawksworth, J. Benjamin Stielow, Thomas J. Walsh, Sean Zhang, Marcus de Melo Teixeira, Daniel Matute, Sybren de Hoog, Dong Wu

**Affiliations:** 1 Department of Respiratory and Critical Care Medicine, Affiliated Hospital of Guangdong Medical University, Zhanjiang, China Affiliated Hospital of Guangdong Medical University Zhanjiang China; 2 Radboudumc/CWZ Center of Expertise for Mycology, Nijmegen, Netherlands Radboudumc/CWZ Center of Expertise for Mycology Nijmegen Netherlands; 3 Foundation Atlas of Clinical Fungi, Hilversum, Netherlands Foundation Atlas of Clinical Fungi Hilversum Netherlands; 4 Department of Parasitology, Faculty of Medicine, Universitas Kristen Indonesia, Jakarta, Indonesia Universitas Kristen Indonesia Jakarta Indonesia; 5 Department of Mycology, Vallabhbhai Patel Chest Institute, University of Delhi, New Delhi, India University of Delhi New Delhi India; 6 Royal Botanic Gardens, Kew, Richmond, Surrey, UK Royal Botanic Gardens, Kew Richmond United Kingdom; 7 Helmholtz Institute for One Health, Greifswald, Germany Helmholtz Institute for One Health Greifswald Germany; 8 Center for Innovative Therapeutics and Diagnostics, Richmond, VA, USA Center for Innovative Therapeutics and Diagnostics Richmond United States of America; 9 University of Maryland School of Medicine, Baltimore, MD, USA University of Maryland School of Medicine Baltimore United States of America; 10 Johns Hopkins University School of Medicine, Baltimore, MD, USA Johns Hopkins University School of Medicine Baltimore United States of America; 11 Núcleo de Medicina Tropical, Campus Universitário Darcy Ribeiro, University of Brasília, Brasília, Brazil University of Brasília Brasília Brazil; 12 Department of Biology, University of North Carolina, Chapel Hill, USA University of North Carolina Chapel Hill United States of America

**Keywords:** Dimorphic pathogens, genealogical concordance, phylogeny, systemic pathogens

## Abstract

The phylogeny of the vertebrate pathogen *Histoplasmacapsulatum* and its varieties was analyzed on the basis of GenBank data, comparing preceding papers that distinguished lineages on the basis of a much smaller dataset, partly dating back two decades. The aim was to establish the predictive value of individual research papers on biodiversity, which eventually may lead to altered nomenclature with large clinical consequences. A total of 1985 sequences of ITS, ARF, OLE and H-anti were downloaded. ITS showed insufficient resolution, and the sequences of the H-anti gene were too short to provide reliable conclusions. Ten major lineages from the seven reports were selected for comparison. Compared to the currently available global data, several earlier studies applied somewhat skewed datasets, biased towards the Americas. Possible separation of Indian and Indonesian lineages were consequently overlooked. Previously distinguished lineages were again recognized, but because of low bootstrap values and extensive genetic exchange, several of these do not deserve species status. No recombination was observed with North American *H.mississippiense* and *H.ohiense*. An African clade (var. duboisii) was individualized. Despite its position in close association with South American clades, histopathology and clinical course of this entity underlines that it has other evolutionary drivers. This might also hold true for the North African donkey disease caused by var. farciminosum, although strains analyzed thus far are indistinguishable from South American strains. On the basis of phylogenetic data, Indian and Indonesian clades are separate, but more clinical data are needed to establish their value as individual species.

## ﻿Introduction

During the past several decades, the taxonomic approach of clinical fungi has undergone a dramatic change from microscopic morphology and phenotype to molecular phylogeny. In general, this has advanced taxonomy, diagnostics, and ecology enormously and has led to abandoning the dual sexual/asexual naming system that was unique to mycology. The speed of accompanying nomenclatural changes, however, has worried clinicians (de Hoog et al. 2023), because nomenclature is the language of communication, the portal to literature, and enables handling of data required for clinical management. Instability of naming is considered detrimental to patient care (Denning 2024). Despite all its advantages, molecular phylogeny as a sole taxonomic approach has a certain degree of intrinsic instability due to its fundamentally relative character, units being compared with each other. Consequently, trees can be affected by taxon sampling (Jantzen et al. 2019). Nomenclatural changes are the product of novel taxonomies, even when based on a single publication, as repeated studies generally are not required. Global representation including all habitats of the species may be difficult, hampering confirmatory analyses. Consequently, studies may feature skewed sets of strains which may not correctly represent the biodiversity of the taxonomic group concerned. Incomplete phylogeny in general is unavoidable, but may be aggravated, for example in sets with an underrepresented global diversity (Rodrigues et al. 2020), a limited number of host plants (Crous et al. 2006), or a selected habitat (Wang et al. 2016). In addition, trees made in the early days of molecular phylogeny mostly applied a single gene for a small number of isolates, while today all studies are multilocus or may apply whole genomes, using a growing number of sequences deposited in GenBank. Subsequent studies of the same fungal group may therefore come to different conclusions within a limited number of years. As nearly all newly discovered medically relevant fungi are opportunistic pathogens (de Hoog et al. 2024), problems in taxonomy of environmental fungi has consequences for medical care.

The re-use of data supplemented by growing amounts of data in GenBank mitigates possible effects of unbalanced strain selection. The share of previously used data becomes larger with every study. It may be assumed that the more recent phylogenies based on using large amounts of data and combining several preceding studies are closer to the natural system than the original ones with scant data. Comparison of original with present-day results then allows estimation of the predictive value of the original trees and taxonomic conclusions. Use of multilocus and genome data also provides deeper insight and estimation of the amount of gene flow between entities that were recognized on the basis of ribosomal sequences only.

One of the much-studied genera of medically relevant fungi is *Histoplasma*. The genus comprises a group of fungi responsible for one of the major systemic diseases, known as histoplasmosis. These fungi produce an environmental phase in soil enriched by bat or pigeon guano located in dry, sheltered places such as caves or abandoned buildings (Gugnani et al. 1994; Taylor et al. 2005; Queiroz-Telles et al. 2017; Gugnani and Denning 2023). Conidia of the environmental phase are easily inhaled, and pulmonary infection may lead to disseminated disease, and possibly to death in patients with impaired cellular immunity (Gómez 2011; Barros et al. 2023). With the AIDS epidemic, the increased use of immunosuppressants in organ transplantation, and global population mobility, the incidence of *Histoplasma* infections has increased rapidly (Adenis et al. 2014; Saullo and Miller 2022).

Several phylogenomic studies (Sepúlveda et al. 2017; Almeida-Silva et al. 2021; Jofre et al. 2022) have provided insight into the evolutionary history of *Histoplasma*. The genus exhibits tremendous genetic diversity (Vite-Garín et al. 2014; Gómez et al. 2019; Rodrigues et al. 2020). Early typing studies suggested the existence of lineages with limited genetic exchange (Carter et al. 2001). Several *Histoplasma* lineages are found to be sufficiently deviating to allow hypotheses of independent virulence levels and host immune evasion strategies (Sepúlveda et al. 2014; Jofre et al. 2022). Some of the genetic clusters have been described as separate cryptic species (Kasuga et al. 1999; Kasuga et al. 2003; Teixeira et al. 2016; Rodrigues et al. 2020).

A logical question is then, whether the early studies based on limited data already had sufficient predictive value for their conclusions to be adopted in practice. The present study aims to retrospectively analyze the taxonomic diversity within *Histoplasma* on the basis of barcoding genes by downloading all available sequences of Internal Transcribed Spacer (ITS), ADP-ribosylation factor (ARF), delta-9 fatty acid desaturase (OLE) and H-antigen precursor (H-anti) from GenBank (date 01-02-2024) and comparing the resulting biodiversity with earlier taxonomic publications using these markers (Kasuga et al. 1999; Kasuga et al. 2003; Adenis et al. 2014; Teixeira et al. 2016; Rodrigues et al. 2020); the conclusions are analyzed with respect to published genome studies (Sepúlveda et al. 2017; Almeida-Silva et al. 2021; Jofre et al. 2022). The selection of these four genes is based on the aforementioned literature, and they represent the largest sets of sequences related to *Histoplasma* in NCBI. The single-gene trees were compared with the results of multilocus studies. Gene-flow between statistically supported clades was verified as a possible criterion of conspecificity. Data of trees and concordance were used to estimate the predictive value of previous classifications, which the caveat that this may have explanations other than sexual recombination. Additionally, by consulting important databases of Index Fungorum, NCBI, Mycobank, and relevant literature, the geographic origins of all *Histoplasma* strains in this study were determined, and relationships between geography and phylogeny were analyzed. This approach allows to establish inasmuch initial classifications based on limited data remain recognizable in larger datasets. Additional, full-genome studies will provide a further level of precision and confidence. This study compares a large set of sequences for a few markers, while genomic studies tend to apply numerous markers for a limited number of strains. Future taxonomy will combine these approaches and integrate these with phenotypic features, leading to integrative taxonomy and ultimate stability. Where necessary, nomenclature of species introduced during *Histoplasma* history were relocated or redefined, aiming at correct naming of every entity to be distinguished.

## ﻿Materials and methods

### ﻿Sequences and previously recognized clades

Sequences of the ITS, ARF, H-anti, and OLE of *Histoplasma* species were downloaded from the NCBI GenBank database or abstracted from genome sequences of related isolates. In view of optimal resolution of phylogenetic relationships, clone correction was applied by retaining only one sequence per gene per isolate. After selection, a total of 879 isolates were included in this study (Suppl. material 1: table S1). Specifically, the sequences involved in the single-gene phylogenetic tree analysis were ITS (*n* = 627), ARF (*n* = 451), OLE (*n* = 491) and H-antigen (*n* = 416), respectively. In multilocus trees, 400 sequences were involved in the ARF / OLE analysis, and 274 sequences were included in four-gene multilocus analyses. The respective phylogenetic trees resulting from these analyses represent the study with the largest number of included strains to date. Strains investigated originated from 47 countries or regions and have a global distribution, with a preponderance (31.74%) of strains from Brazil. Original names and numbers were maintained as deposited in GenBank to enhance recognition of strains. A search of original literature revealed living type materials for all names formally introduced in *Histoplasma*. For several, living strains proved to be extant but not always available for analysis of all genes covered in this study. Strains were then attributed to their clades on the basis of data available in GenBank, while in some cases the missing genes were sequenced in-house or abstracted from genomes according to the original papers.

Ten clades maintained at phylogenetic species level in seven earlier studies were selected as guidelines in this study (Kasuga et al. 1999; Kasuga et al. 2003; Teixeira et al. 2016; Sepúlveda et al. 2017; Rodrigues et al. 2020; Almeida-Silva et al. 2021; Jofre et al. 2022). The entities have been indicated in these papers as NAm-1, NAm-2, Africa, LAm-A (LAm-A1 and LAm-A2), LAm-B (LAm-B1 and LAm-B2), LAm-C, LAm-D, LAm-E, RJ and India, and an additional Indonesia clade identified in this study.

### ﻿DNA extraction and amplification

In-house sequenced strains (CBS 215.53) were grown on malt extract agar (MEA) for 14 days. Approximately 1 cm^**2**^ of material was added to a screw-capped tube containing 490 µL CTAB-buffer (2% cetyltrimethylammonium bromide, 100 mM Tris-HCl, 20 mM EDTA, 1.4 M NaCl) and 6–10 acid-washed glass beads. The above procedures were performed inside a class II biological safety cabinet under conditions of biosafety level 3 (BSL3) containment. Ten units of proteinase K were added to the mixture and vortexed with a MOBIO vortex for a few min. Tubes were incubated at 60 °C for 60 min. After incubation, the tubes were again vortexed and 500 µL of chloroform: isoamyl alcohol (24 : 1) were added followed by shaking for 2 min. Tubes were spun at 14,000 r.p.m. in a microfuge for 10 min and the upper layer was collected in new sterile tubes with 0.55 volume ice-cold iso-propanol and spun again. The pellets were washed with 70% ethanol, air-dried and re-suspended in 50 µL TE buffer. DNA amplification was performed for ARF and H-antigen. Primers used for amplification and sequencing of ARF were ARF1 and ARF2, for H-anti these were H-anti3 and H-anti4 (Kasuga et al. 2003). The amplifications were carried out in 50 μL reaction mixtures [10 μL PCR buffer, 1 μL dNTPs, 1 μM of each primer (10 pmol), 0.5 μL *Taq* polymerase, 0.5 μL DNA and 36 μL water].

### ﻿Alignment and phylogenetic analysis

Sequences of ITS, ARF, OLE, H-anti of related isolates were edited using BIOEDIT v7.2 (Hall 1999). Alignments were made by MAFFT v7 (http://mafft.cbrc.jp/) and optimized manually using MEGA v7.2 (Kumar et al. 2012) and BIOEDIT v7.2. Missing data for partial or complete sequences in some taxa were coded as ‘missing’ (Wiens 2006). To address the phylogenetic relationships among taxa, Maximum Likelihood (ML) and Bayesian inference (BI) algorithms were used (http://www.phylo.org/). For better comparison, we also used the IQ-TREE (http://www.iqtree.org/doc/Concordance-Factor) web server and applied the Maximum Likelihood algorithm to construct the phylogenetic trees. One strain with a long branch in all trees was selected as outgroup. Trees were edited using TREEVIEW v1.6.6 and completed with Adobe ILLUSTRATOR CS v5. The bootstrap of 11 clades in different phylogenetic trees are shown in Table 1.

**Table 1.** Bootstrap values of 11 clades in different phylogenetic trees. All clades were recognized and reported in previous studies. ‘No’ means the group did not form a separate clade.

	ITS	ARF	OLE	H-anti	Two genes	Multi-gene
LAm A	no, no, no	no, no, no	no, no, no	no, no, no	no, no, no	no, no, no
LAm B	no, no, no	no, no, no	no, no, no	54, 96, 84	no, no, no	no, no, no
LAm C	no, no, no	68, 98, 84	35, 84, 66	no, no, no	48, 96, 96	55, 97, 64
LAm D	no, no, no	no, no, no	no, no, no	no, no, no	no, no, no	no, no, no
LAm E	no, no, no	no, no, no	no, no, no	no, no, no	no, no, no	no, no, no
RJ	no, no, no	no, no, no	no, no, no	no, no, no	no, no, no	no, no, no
NAm 1	77, 87, 100	97, 99, 100	99, 100, 100	no, no, no	100, 100, 100	100, 100, 100
NAm 2	98, 99, 100	98, 99, 100	96, 100, 100	no, no, no	100, 100, 100	100, 100, 100
Africa	98, 99, 100	95, 99, 100	97, 99, 100	no, no, no	100, 100, 100	99, 98, 100
India	87, 100, 100	95, 99, 100	80, 98, 100	no, no, no	96, 98, 100	81, 97, 100
Indonesia	no, no, no	76, 99, 99	87, 98, 100	no, no, no	99, 99, 100	100, 100, 100

### ﻿Genetic population analysis

The haplotype diversities were estimated based on two genes, ARF and OLE. Haplotype networks for the genes analyzed were plotted using the geneHapR library v1.1.9 (Zhang et al. 2023). Haplotype networks were built using the same sequences as those used for constructing the ARF and OLE phylogenetic trees. Due to the low diversity of ITS and the short sequences of H-anti, these two genes were excluded from the haplotype network analysis. We used seven different colored spheres to represent seven different categories: six categories represent six clades *H.capsulatum*, *H.* (var.) *duboisii*, *H.mississippiense*, *H.ohiense*, India and Indonesia. The seventh represents strains from Panama, as these include the type strain of *Histoplasmacapsulatum*. The size of each sphere is proportional to the number of strains belonging to that haplotype. The geographic of each clades was calculated and shown in the maps.

### ﻿Concordance for phylogenetic species recognition

To assess the concordance of lineages among different genes of studied strains, we designed two sets of analyses. The first set involved a selection of strains all having sequences for four genes, and constructing individual single-gene phylogenetic trees. These trees were pairwise compared using a custom-designed encoding program (ITS vs. ARF, ITS vs. OLE, ARF vs. OLE). The combined results were then used to determine potential phylogenetic species. In the second set of analyses, only strains previously defined as LAm-A, LAm-B, LAm-C, LAm-D, LAm-E, RJ, NAm-1, NAm-2, Africa, Indonesia and India were retained. Individual phylogenetic trees for the ARF and OLE genes were constructed, and their lineage relationships were analyzed using the custom-designed encoding program.

## ﻿Results

### ﻿Tree comparison

To enhance the reliability of the phylogenetic tree in this study, three tree models were applied: a maximum likelihood (ML) tree based on the CIPRES platform, ML trees based on the IQ-TREE platform, and Bayesian trees based on the CIPRES platform. The results indicate that the topologies of these three trees are highly consistent, with the main differences in branch support values. The ML tree generated with IQ-TREE in general yielded highest branch support, with some branches that did not receive significant support in the other trees showing support rates above 90% in the IQ-TREE ML tree. In the following parts, only the ML tree based on the CIPRES platform is presented, with branch support values of these three trees listed in the order of ML (CIPRES), ML (IQ-TREE), and Bayes (CIPRES).

### ﻿ITS

Through a search in GenBank and extraction from genomes, we collected a total of 627 ITS sequences deposited under the generic name *Histoplasma* or *Ajellomyces*, after removing some duplicate sequences, 499 sequences were retained for analysis. This extensive data set comprehensively represents all currently known taxa in *Histoplasma*. Aligned ITS sequences were 470 bp long with the following base frequencies: pi(A) = 0.186443, pi(T) = 0.204952, pi(C) = 0.309767, pi(G) = 0.298837. ML tree generated by CIPRES was shown (Suppl. material 1: fig. S1, table S1). Sequences of strains CADAM, AC02, and RS36 stood out in a long branch which could not be explained by their low number (n = 7) of SNPs. The results indicate that most ITS sequences of *Histoplasma* share the same sequence and few differences between strains. Four supported clades contained reference strains of described phylogenetic species (NAm-1, NAm-2, Africa, India). In the ITS tree, nine strains form a distinct clade (NAm-2, BS 98%, BS 99%, PI 100%) with 11 additional strains from North America and two strains from South America. CBS 136.72 was described as ex-type strain of *Histoplasmacapsulatum*. G217B was described as type strain of *Histoplasmaohiense*. Both strains clustered together in this clade. Similarly, nine strains cluster together in a clade referred to as NAm-1 with support rate (BS 77%, BS 87%, PI 100%); this clade includes 11 further U.S.A. strains, plus 5 strains from Iraq and 1 strain from Austria. A third supported clade (BS 98%, BS 99%, PI 100%) comprises strains exclusively from Africa. Eleven strains from India clustered together into a subclade with support rate (BS 87%, BS 100%, PI 100%). The strains previously grouped as the phylogenetic species LAm did not aggregate in supported clades. Strains formerly indicated as RJ, LAm-A, LAm-A1, and LAm-A2 were found scattered throughout the tree. Similarly, strains referred to as LAm-B, LAm-B1, and LAm-B2 were dispersed in several clades of the tree. The three clades reported by Rodrigues et al. (2020), i.e. LAm-C, LAm-D, and LAm-E were intermixed with strains from the LAm-A and RJ group. Twelve isolates from India and 9 isolates from Indonesia clustered together in a clade containing strains all originating from Asia, but with low bootstrap support.

Groups with identical sequences, even when lacking statistical support, mostly originated from the same geographic region. The upper half of ITS tree comprising 70 identical sequences contains only five strains from outside South America, i.e. IFM 41330 from Japan, and four strains from North America. The backbone of the lower part of the tree also contains an overabundance of identical sequences from South America(*n* = 83), but also from other continents. Clusters of strains that deviated by a few SNPs from the main groups, despite absence of statistical support, nearly always originated from a single country.

### ﻿ARF

**Figure 1.**
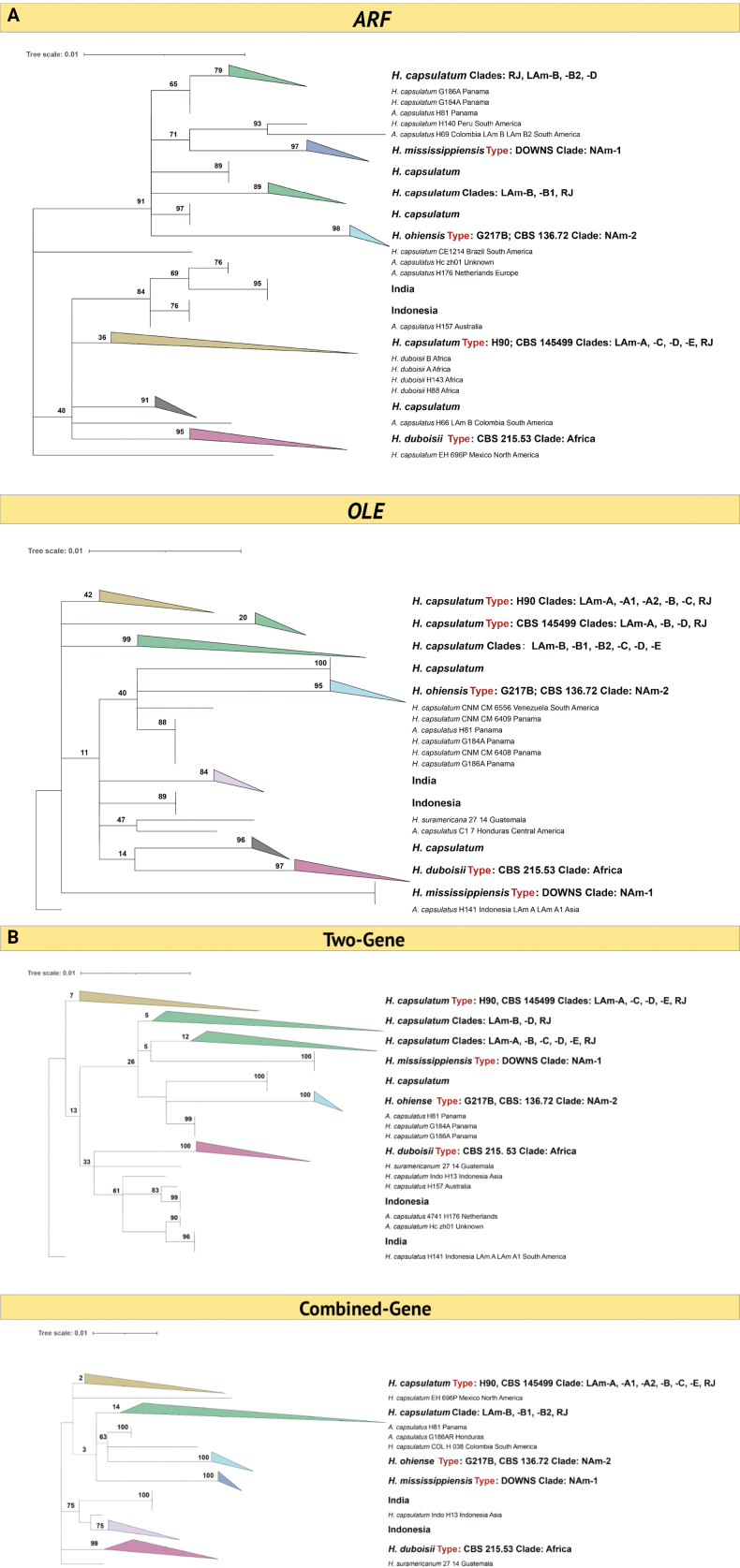
Collapsed phylogenetic tree of *Histoplasma* based on ARF, OLE, two genes (ARF and OLE) or multilocus sequences (ITS, ARF, OLE and H-anti), obtained by maximum likelihood. All the bootstrap are shown. Different colors represent different clades. Single clade with long branch were selected as outgroup. **A** ARF tree and OLE tree; **B** two-gene tree and Combined-Gene.

Aligned ARF sequences were 418 bp, with the following base frequencies: pi(A) = 0.298862, pi(T)= 0.257376, pi(C) = 0.239235, pi(G) = 0.204527. In the ML tree (Fig. 1A; Suppl. material 1: fig. S2, table S1), numerous subclusters that deviate in a single SNP are nearly always composed of strains from a single or from adjacent countries. One cluster with 66% BS contains twelve strains from Europe and one strain H90 from Egypt, which was described as type strain of Histoplasmacapsulatumvar.farciminosum. Strains from Central America (Honduras, Mexico) are relatively variable, belonging to several small clusters. The reference strains of previously distinguished taxa are found in an African clade (BS 95%, BS 99%, PI 100%), and strains referred to as NAm-1 (BS 97%, BS 99%, PI 100%) and NAm-2 (BS 98%, BS 99%, PI 100%). A clade with support rate (BS 84%, BS 99%, PI 100%) with non-South American strains contains three subclusters: one subclade contains strains exclusively from India (BS 95%, BS 99%, PI 100%), one subclade contains strains exclusively from Indonesia (BS 76%, BS 99%, PI 99%).

Comparing the current ARF tree with previously recognized groups, a group referred to as LAm-A has been recognized as an independent clade (Kasuga et al. 2003; Rodrigues et al. 2020). Teixeira et al. (2016) divided LAm-A into two subclades, LAm-A1 and LAm-A2. In the current ARF tree, the strains of these subclades do not show significant differences; instead, they were scattered in one big clade with other LAm strains. LAm-B was accepted as a phylogenetic species in the studies of Kasuga et al. (2003) and Rodrigues et al. (2020). Teixeira et al. (2016) distinguished two subclades, LAm-B1 and LAm-B2. In the current ARF data, the bipartition of LAm-B was recognized: a subclade (BS 89%, BS 98%, PI 100%) contained three RJ strains, 16 LAm-B or LAm-B1 strains, in addition to some other isolates, and a subclade (BS 79%, BS 98%, PI 92%) with 12 strains of LAm-B or LAm-B2 groups, 13 strains of RJ group, 2 strains of LAm-D, and some other strains. Exceptions were strains H66 and H69, which were considered to belong to LAm-B but were found to be forming independent clades rather than clustering into any of the LAm-B clades. All the strains in these clades originated from South America.

Teixeira et al. (2016) recognized a clade ‘RJ’ as a possible cryptic species, of which isolates of the LAm-A group were collected in Rio de Janeiro and São Paulo states in Brazil. This clade was confirmed by Rodrigues et al. (2020). However, in the current ARF tree, strains identified as RJ were mixed with other Latin American strains, scattered across different clades rather than forming a distinct clade. The reason might be that ‘RJ’ indicates a collection, with not all strains originating from the Rio de Janeiro area.

Rodrigues et al. (2020) recognized several further cryptic entities, named LAm-C, -D and -E. All strains referred to as LAm-C clustered together in a major clade. Despite the support rates not being high (BS 60%, BS 98%, PI 84%), this clade contained strains originating exclusively from Brazil. The strains of LAm-D were divided into two parts. Two strains clustered with LAm-B (described above), while three strains were close to LAm-A strains. LAm-E comprised only two strains, which were close to each other but did not form an independent clade. Consequently, the ARF data did not support these Latin American groups.

### ﻿OLE and H-antigen

The OLE gene alignment contained 491 sequences. Aligned OLE sequences were 407 bp, with the following base frequencies: pi(A) = 0.226902, pi(T) = 0.269648, pi(C) = 0.281335, pi(G) = 0.222114. An African and two North American clades were recognized, but within the sequences from Latin America no supported clades were found, strains belonging to subclades with other genes being intermixed, without forming distinct clades(Fig. 1A; Suppl. material 1: fig. S3, table S1). Twenty strains from Africa all clustered in a clade with support rate (BS 97%, BS 99%, PI 100%), containing not a single strain from another continent. Fourteen strains from the U.S.A, and one from Austria formed a clade previously referred to as NAm-1 (Kasuga et al. 1999; Kasuga et al. 2003; Teixeira et al. 2016; Rodrigues et al. 2020), with support rate (BS 99%, BS 100%, PI 100%). Twenty one from the U.S.A. and three from Colombia formed the NAm-2 branch (BS 96%, BS 100%, PI 100%). One of these Colombia strains (1986) was described as *Histoplasmasuramericanum* by Sepúlveda et al. (2017). Additionally, 12 strains from Indonesia clustered with one strain from Australia in a clade with support rate (BS 87%, BS 98%, PI 100%); 16 strains from India, one strain from The Netherlands (probably concerning an immigrant) and one unknown strain formed a sister clade next to the Indonesian strains (BS 80%, BS 98%, PI 100%). A highly supported clade (BS 99%, BS 100%, PI 100%) comprised 27 strains, including those isolates of groups previously referred to as LAm-A, LAm-B, LAm-D, and RJ, the ex-type strain of *H.suramericanum* MZ5 also cluster in this clade (Teixeira et al. 2016; Sepúlveda et al. 2017; Rodrigues et al. 2020). The origins of these strains are diverse, spanning five countries from Brazil to Honduras.

The H-antigen gene tree contained 416 sequences (Suppl. material 1: fig. S4), with an alignment length of 223 bp. However, due to the short length of the sequences and the limited number of informative sites, this gene provided insufficient resolution for the purpose of the present paper.

### ﻿Multilocus

Concatenated two-gene trees were made (n = 400, Fig. 1B; Suppl. material 1: fig. S5, table S1), and a multilocus tree using all four genes under study (n = 274, Fig. 1B; Suppl. material 1: fig. S6, table S1). The alignment of ARF + OLE was 826 bp long, with the following base frequencies: pi (A) = 0.264853, pi (T) = 0.262417, pi (C) = 0.259700, pi (G) = 0.213030. The alignment of the multilocus tree was 1467 bp long, with the following base frequencies: pi (A) = 0.237208, pi (T) = 0.245648, pi (C) = 0.263872, pi (G) = 0.253273.

In the ARF-OLE phylogenetic tree, LAm-C (BS 48%, BS 96%, PI 96%), India (BS 96%, BS 98%, PI 100%), Indonesia (BS 99%, BS 99%, PI 100%), Africa (BS 100%, BS 100%, PI 100%), NAm-1 (BS 100%, BS 100%, PI 100%), and NAm-2 (BS 100%, BS 100%, PI 100%) formed distinct clades. All LAm-A strains, two strains of LAm-E, five strains of LAm-D, and some RJ strains were found scattered throughout the tree, without forming supported clades. Most strains of LAm-B were distributed among two subclades, with low support values. One supported clade (BS 99%, BS 99%, PI 100%) contained 12 strains from Indonesia and one from Australia. 15 strains from India form a single clade (BS 96%, BS 98%, PI 100%).

Similar to the ARF-OLE tree, the multilocus tree revealed supported clades for LAm-C (BS 55%, BS 97%, PI 64%), India (BS 81%, BS 97%, PI 100%), Indonesia (BS 100%, BS 100%, PI 100%), Africa (BS 99%, BS 98%, PI 100%), NAm-1 (BS 100%, BS 100%, PI 100%) and NAm-2 (BS 100%, BS 100%, PI 100%). The strains from South and Central America did not form distinct clades; instead, they were dispersed in unsupported subgroups or intermixed with each other. Consequently, the multilocus tree does not support the distinction of LAm-A, LAm-B, LAm-C, LAm-D, LAm-E and RJ as phylogenetic species.

### ﻿Concordance of clades

**Figure 2.**
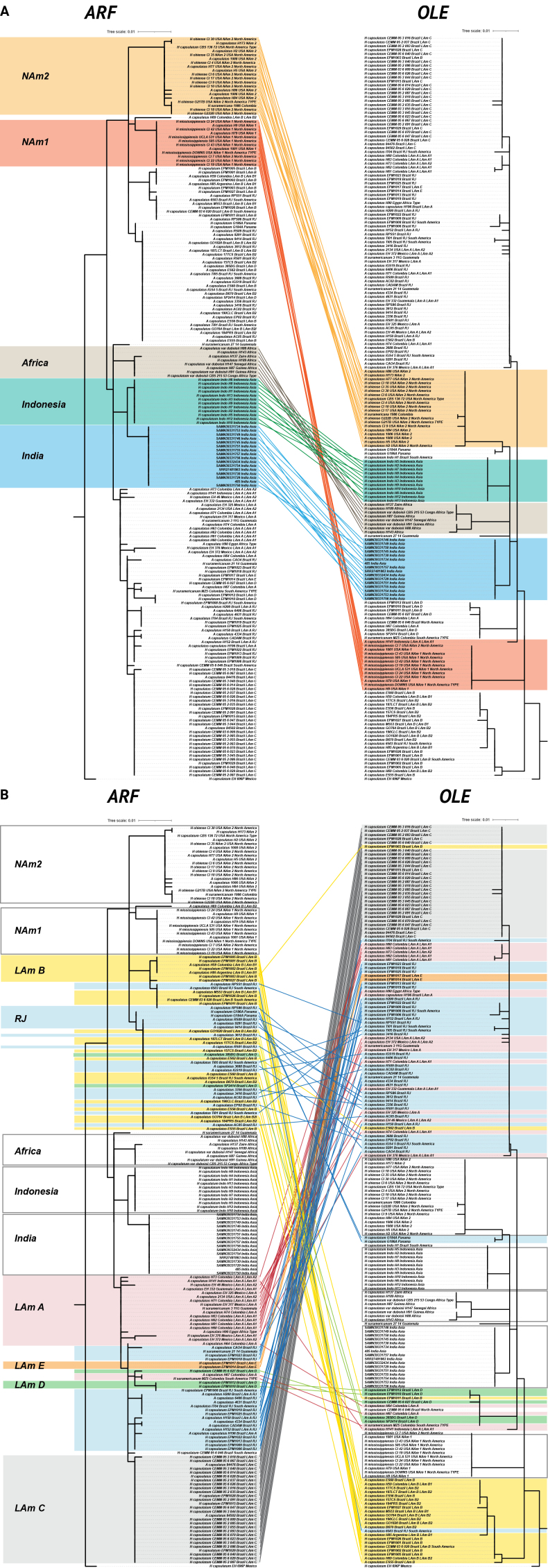
Genealogical concordance of analysed sequences of ARF and OLE. Tanglegram comparisons were applied to analysis of gene exchange in these species. **A** Clades did not show phylogenetic consistency in ARF 2 and OLE 2; **B** clades show phylogenetic consistency in ARF 2 and OLE 2.

Based on the analyses of the above single-gene and multilocus phylogenetic trees, we identified two genes, ARF and OLE, which most effectively resolved the phylogenetic relationships within the genus *Histoplasma*. We then analyzed the concordance between these two genes by comparing the compositions of supported clades. The trees resulting from this analysis were labeled as AFR2 and OLE2 to differentiate them from the previously mentioned single-gene tree. To clarify the results, only 190 strains defined as 10 clades (Africa, NAm-1, NAm-2, LAm A-E, RJ, India) by previous studies, and an additional Indonesia clade identified in this study, were retained for concordance analysis of these two genes (ARF, Fig. 2A; OLE, Fig. 2B; Table 2). Twenty-eight strains of LAm-C cluster in separate clades with bootstrap values of 73% and 48%, respectively. Five strains of LAm-D cluster together in OLE2 (Suppl. material 1: fig. S7), but only three strains cluster together in ARF2 (Suppl. material 1: fig. S8), indicating that the lineage relationships are consistent for only three strains of LAm-D. The strains of groups LAm-A, LAm-B, LAm-E and RJ did not show phylogenetic consistency in ARF 2 and OLE 2. Some of the clusters were interspersed with each other, suggesting gene flow. For the Africa clade, six strains show consistent lineage relationships, while the remaining two African strains, H88 and H143, do not form a clade with other African strains but are located adjacent to the African clade in ARF 2 although included in the Africa clade in the OLE 2. Other clades, NAm-1, NAm-2, India and Indonesia all were concordant in these two genes, suggesting reproductive isolation. We also performed pairwise comparisons of the four single-gene phylogenetic trees. The results were similar to those of the two-gene comparisons, and therefore are not displayed.

**Table 2.** Strains defined as 10 clades (Africa, NAm-1, NAm-2, LAm A-E, RJ, India) by previous studies, and an additional Indonesia clade identified in this study, were retained for genealogic concordance analysis of ARF and OLE genes. ‘No’ means the strain did not fall in any separate clade.

Name	Previous identified Clades	Strain	ARF	OLE	Country
*A.capsulatus*	Africa	H87	AF072336	Tree base #1063	Africa
*H.capsulatum*	Africa	H147	Tree base #1063	Tree base #1063	Senegal
*H.capsulatum*	Africa	H91	JX443637	JX458513	Guinea
*A.capsulatus*	Africa	H189	Tree base #1063	Tree base #1063	Unknown
*A.capsulatus*	Africa	H137	AF072337	Tree base #1063	Zaire
*H.capsulatum*	Africa	CBS 215.53	PQ055824	KX646107	Congo
*H.capsulatum*	Africa	H143	Tree base #1063	FJ435632	Africa
*H.capsulatum*	Africa	H88	AF072335	FJ435631	Africa
*H.capsulatum*	Panama	G184A	abstracted from genome	abstracted from genome	Panama
*H.capsulatum*	Panama	G186A	abstracted from genome	abstracted from genome	Panama
*Histoplasma* sp.	India	SAMN39331734	abstracted from genome	abstracted from genome	India
*Histoplasma* sp.	India	SAMN39331745	abstracted from genome	abstracted from genome	India
*Histoplasma* sp.	India	SAMN39331746	abstracted from genome	abstracted from genome	India
*Histoplasma* sp.	India	SAMN39331749	abstracted from genome	abstracted from genome	India
*Histoplasma* sp.	India	SAMN39331750	abstracted from genome	abstracted from genome	India
*Histoplasma* sp.	India	SAMN39331751	abstracted from genome	abstracted from genome	India
*Histoplasma* sp.	India	SAMN39331753	abstracted from genome	abstracted from genome	India
*Histoplasma* sp.	India	SAMN39331754	abstracted from genome	abstracted from genome	India
*Histoplasma* sp.	India	SAMN39331755	abstracted from genome	abstracted from genome	India
*Histoplasma* sp.	India	SAMN39331756	abstracted from genome	abstracted from genome	India
*Histoplasma* sp.	India	SAMN39331757	abstracted from genome	abstracted from genome	India
*Histoplasma* sp.	India	SAMN39332434	abstracted from genome	abstracted from genome	India
*Histoplasma* sp.	India	SRR27481863	abstracted from genome	abstracted from genome	India
*Histoplasma* sp.	India	485	abstracted from genome	abstracted from genome	India
*Histoplasma* sp.	India	SAMN39331729	abstracted from genome	abstracted from genome	India
*Histoplasma* sp.	India	SAMN39331730	abstracted from genome	abstracted from genome	India
*Histoplasma* sp.	Indonesia	Indo_H1	MN637625	MN637651	Indonesia
*Histoplasma* sp.	Indonesia	Indo_H10	MN637634	MN637660	Indonesia
*Histoplasma* sp.	Indonesia	Indo_H12	MN637635	MN637662	Indonesia
*Histoplasma* sp.	Indonesia	Indo_H13	MN637636	MN637663	Indonesia
*Histoplasma* sp.	Indonesia	Indo_H2	MN637626	MN637652	Indonesia
*Histoplasma* sp.	Indonesia	Indo_H3	MN637627	MN637653	Indonesia
*Histoplasma* sp.	Indonesia	Indo_H4	MN637628	MN637654	Indonesia
*Histoplasma* sp.	Indonesia	Indo_H5	MN637629	MN637655	Indonesia
*Histoplasma* sp.	Indonesia	Indo_H6	MN637630	MN637656	Indonesia
*Histoplasma* sp.	Indonesia	Indo_H7	MN637631	MN637657	Indonesia
*Histoplasma* sp.	Indonesia	Indo_H8	MN637632	MN637658	Indonesia
*Histoplasma* sp.	Indonesia	Indo_H9	MN637633	MN637659	Indonesia
*A.capsulatus*	LAm A	H196	Tree base #1063	Tree base #1063	Brazil
*A.capsulatus*	LAm A	H64	AF072356	Tree base #1063	Colombia
*A.capsulatus*	LAm A	H67	JX443633	Tree base #1063	Colombia
*H.capsulatum*	LAm A	EH_317	AF495591	AF495593	Mexico
*A.capsulatus*	LAm A	EH_325	Tree base #1063	Tree base #1063	Mexico
*A.capsulatus*	LAm A/LAm A1	H141	Tree base #1063	Tree base #1063	Indonesia
*A.capsulatus*	LAm A/LAm A1	H60	AF072352	JX458506	Colombia
*A.capsulatus*	LAm A/LAm A1	H62	AF072354	Tree base #1063	Colombia
*H.capsulatum*	LAm A/LAm A1	EH_376	AF495611	AF495613	Mexico
*A.capsulatus*	LAm A/LAm A1	EH_332	Tree base #1063	Tree base #1063	Guatemala
*A.capsulatus*	LAm A/LAm A1	H63	AF072355	Tree base #1063	Colombia
*A.capsulatus*	LAm A/LAm A1	H61	AF072353	JX458507	Colombia
*A.capsulatus*	LAm A/LAm A2	H74	AF072360	Tree base #1063	Colombia
*A.capsulatus*	LAm A/LAm A2	H71	AF072358	JX458511	Colombia
*A.capsulatus*	LAm A/LAm A2	EH_372	AF495595	AF495597	Mexico
*H.capsulatum*	LAm A/LAm A2	EH_46	Tree base #1063	Tree base #1063	Mexico
*A.capsulatus*	LAm A/LAm A2	H73	AF072359	Tree base #1063	Colombia
*A.capsulatus*	LAm A/LAm A2	2134	JX443630	JX458503	USA
*A.capsulatus*	LAm A/RJ	H152	Tree base #1063	Tree base #1063	Brazil
*A.capsulatus*	LAm A/RJ	H200	Tree base #1063	Tree base #1063	Brazil
*A.capsulatus*	LAm A/RJ	H150	Tree base #1063	Tree base #1063	Brazil
*H.capsulatum*	LAm B	CEMM_03_6_020	MK893575	MK893779	Brazil
*H.capsulatum*	LAm B	EPM1001	MK893647	MK893547	Brazil
*H.capsulatum*	LAm B	EPM1003	MK893649	MK893854	Brazil
*H.capsulatum*	LAm B	EPM1011	MK893657	MK893862	Brazil
*H.capsulatum*	LAm B	EPM1026	MK893672	MK893878	Brazil
*H.capsulatum*	LAm B	EPM1027	MK893673	MK893878	Brazil
*A.capsulatus*	LAm B	ES55	GU320868	GU320996	Brazil
*A.capsulatus*	LAm B	ES56	GU320869	GU320997	Brazil
*A.capsulatus*	LAm B	ES60	GU320870	GU320998	Brazil
*A.capsulatus*	LAm B	ES62	GU320871	GU320999	Brazil
*H.capsulatum*	LAm B	EPM1002	MK893648	MK893853	Brazil
*H.capsulatum*	LAm B	EPM1005	MK893651	MK893856	Brazil
*A.capsulatus*	LAm B/LAm B1	H59	JX443631	JX458505	Colombia
*A.capsulatus*	LAm B/LAm B1	MS53	GU320847	GU321036	Brazil
*A.capsulatus*	LAm B/LAm B1	H85	AF072367	Tree base #1063	Argentina
*A.capsulatus*	LAm B/LAm B2	187LCT	GU320876	GU321010	Brazil
*A.capsulatus*	LAm B/LAm B2	GO1820	GU320864	GU320992	Brazil
*A.capsulatus*	LAm B/LAm B2	GO764	GU320863	GU320991	Brazil
*A.capsulatus*	LAm B/LAm B2	H69	AF072364	JX458510	Colombia
*A.capsulatus*	LAm B2	157CS	GU320875	GU321009	Brazil
*A.capsulatus*	LAm B2	177CS	GU320884	GU321037	Brazil
*A.capsulatus*	LAm B2	184PRS	GU320883	GU321011	Brazil
*A.capsulatus*	LAm B2	190CLC	GU320877	GU320987	Brazil
*A.capsulatus*	LAm B2	B670	GU320882	GU321035	Brazil
*A.capsulatus*	LAm C	84476	GU320841	GU321008	Brazil
*A.capsulatus*	LAm C	84502	GU320840	GU321006	Brazil
*H.capsulatum*	LAm C	CEMM_03_3_055	MK893645	MK893850	Brazil
*H.capsulatum*	LAm C	CEMM_03_6_009	MK893597	MK893801	Brazil
*H.capsulatum*	LAm C	CEMM_05_2_035	MK893576	MK893780	Brazil
*A.capsulatus*	LAm C	CEMM_05_2_037	MK893594	MK893798	Brazil
*H.capsulatum*	LAm C	CEMM_05_2_085	MK893603	MK893807	Brazil
*H.capsulatum*	LAm C	CEMM_05_2_086	MK893581	MK893785	Brazil
*H.capsulatum*	LAm C	CEMM_05_2_087	MK893579	MK893783	Brazil
*H.capsulatum*	LAm C	CEMM_05_2_091	MK893599	MK893803	Brazil
*H.capsulatum*	LAm C	CEMM_05_2_093	MK893582	MK893786	Brazil
*H.capsulatum*	LAm C	CEMM_05_3_016	MK893596	MK893800	Brazil
*H.capsulatum*	LAm C	CEMM_05_3_018	MK893637	MK893842	Brazil
*H.capsulatum*	LAm C	CEMM_05_3_040	MK893611	MK893815	Brazil
*H.capsulatum*	LAm C	CEMM_05_3_044	MK893578	MK893782	Brazil
*H.capsulatum*	LAm C	CEMM_05_3_045	MK893587	MK893791	Brazil
*H.capsulatum*	LAm C	CEMM_05_6_014	MK893608	MK893812	Brazil
*H.capsulatum*	LAm C	CEMM_05_6_023	MK893600	MK893804	Brazil
*H.capsulatum*	LAm C	CEMM_05_6_026	MK893604	MK893808	Brazil
*H.capsulatum*	LAm C	CEMM_05_6_029	MK893590	MK893794	Brazil
*H.capsulatum*	LAm C	CEMM_05_6_040	MK893624	MK893828	Brazil
*H.capsulatum*	LAm C	CEMM_05_6_047	MK893638	MK893843	Brazil
*H.capsulatum*	LAm C	CEMM_05_6_067	MK893640	MK893845	Brazil
*H.capsulatum*	LAm C	CEMM_05_6_070	MK893643	MK893848	Brazil
*H.capsulatum*	LAm C	EPM1015	MK893661	MK893866	Brazil
*H.capsulatum*	LAm C	EPM1028	MK893674	MK893879	Brazil
*H.capsulatum*	LAm C	EPM1029	MK893675	MK893880	Brazil
*H.capsulatum*	LAm C	CEMM_05_6_028	MK893577	MK893781	Brazil
*A.capsulatus*	LAm D	385BG	GU320865	GU320993	Brazil
*H.capsulatum*	LAm D	CEMM_05_6_027	MK893614	MK893818	Brazil
*H.capsulatum*	LAm D	EPM1008	MK893654	MK893859	Brazil
*H.capsulatum*	LAm D	EPM1010	MK893656	MK893861	Brazil
*H.capsulatum*	LAm D	EPM1012	MK893658	MK893863	Brazil
*A.capsulatus*	LAm D	SP2414	GU320867	GU320995	Brazil
*H.capsulatum*	LAm D	CEMM_05_6_046	MK893628	MK893832	Brazil
*H.capsulatum*	LAm E	EPM1014	MK893660	MK893865	Brazil
*H.capsulatum*	LAm E	EPM1017	MK893663	MK893868	Brazil
*A.capsulatus*	NAm 1	H9	AF072350	Tree base #1063	USA
*A.capsulatus*	NAm 1	H79	AF072349	Tree base #1063	USA
*A.capsulatus*	NAm 1	1001	JX443624	JX458499	USA
*H.mississippiensis*	NAm 1	505	abstracted from genome	abstracted from genome	USA
*H.mississippiensis*	NAm 1	CI_19	abstracted from genome	abstracted from genome	USA
*H.mississippiensis*	NAm 1	CI_22	abstracted from genome	abstracted from genome	USA
*H.mississippiensis*	NAm 1	CI_24	abstracted from genome	abstracted from genome	USA
*H.mississippiensis*	NAm 1	CI_42	abstracted from genome	abstracted from genome	USA
*H.mississippiensis*	NAm 1	CI_43	abstracted from genome	abstracted from genome	USA
*H.mississippiensis*	NAm 1	CI_7	abstracted from genome	abstracted from genome	USA
*H.mississippiensis*	NAm 1	DOWNS	abstracted from genome	abstracted from genome	USA
*H.mississippiensis*	NAm 1	UCLA_531	abstracted from genome	abstracted from genome	USA
*A.capsulatus*	NAm 2	H2	AF072339	Tree base #1063	USA
*A.capsulatus*	NAm 2	H5	AF072340	Tree base #1063	USA
*A.capsulatus*	NAm 2	H84	AF072345	Tree base #1063	USA
*A.capsulatus*	NAm 2	H86	AF072346	Tree base #1063	USA
*A.capsulatus*	NAm 2	H173	Tree base #1063	Tree base #1063	Unknown
*A.capsulatus*	NAm 2	1006	JX443627	JX458501	USA
*A.capsulatus*	NAm 2	1008	JX443628	JX458502	USA
*H.capsulatum*	NAm 2	CBS_136_72	OM837779	KX646110	USA
*A.capsulatus*	NAm 2	H77	AF072344	Tree base #1063	USA
*H.ohiense*	NAm 2	CI_10	abstracted from genome	abstracted from genome	USA
*H.ohiense*	NAm 2	CI_17	abstracted from genome	abstracted from genome	USA
*H.ohiense*	NAm 2	CI_18	abstracted from genome	abstracted from genome	USA
*H.ohiense*	NAm 2	CI_30	abstracted from genome	abstracted from genome	USA
*H.ohiense*	NAm 2	CI_35	abstracted from genome	abstracted from genome	USA
*H.ohiense*	NAm 2	CI_4	abstracted from genome	abstracted from genome	USA
*H.ohiense*	NAm 2	CI_6	abstracted from genome	abstracted from genome	USA
*H.ohiense*	NAm 2	CI_9	abstracted from genome	abstracted from genome	USA
*H.ohiense*	NAm 2	G217B	abstracted from genome	abstracted from genome	USA
*H.ohiense*	NAm 2	G222B	abstracted from genome	abstracted from genome	USA
*A.capsulatus*	No	H90	AF072351	Tree base #1063	Egypt
*H.capsulatum*	No	EH_696P	KT601380	KT601417.1	Mexico
*H.suramericanum*	no	1986	abstracted from genome	abstracted from genome	Colombia
*H.suramericanum*	no	21_14	abstracted from genome	abstracted from genome	Guatemala
*H.suramericanum*	no	27_14	abstracted from genome	abstracted from genome	Guatemala
*H.suramericanum*	no	3_11G	abstracted from genome	abstracted from genome	Guatemala
*H.suramericanum*	no	MZ5	abstracted from genome	abstracted from genome	Colombia
*A.capsulatus*	RJ	3416	GU320880	GU321023	Brazil
*A.capsulatus*	RJ	6406	GU320837	GU321034	Brazil
*A.capsulatus*	RJ	9414	GU320873	GU321004	Brazil
*A.capsulatus*	RJ	AC02	GU320858	GU321013	Brazil
*A.capsulatus*	RJ	AC05	GU320859	GU321021	Brazil
*A.capsulatus*	RJ	CADAM	GU320843	GU321014	Brazil
*A.capsulatus*	RJ	CAO4	GU320844	GU321022	Brazil
*A.capsulatus*	RJ	EP02	GU320878	GU321015	Brazil
*H.capsulatum*	RJ	EPM1006	MK893652	MK893857	Brazil
*H.capsulatum*	RJ	EPM1009	MK893655	MK893860	Brazil
*H.capsulatum*	RJ	EPM1013	MK893659	MK893864	Brazil
*H.capsulatum*	RJ	EPM1019	MK893665	MK893871	Brazil
*H.capsulatum*	RJ	EPM1022	MK893668	MK893873	Brazil
*H.capsulatum*	RJ	EPM1023	MK893669	MK893874	Brazil
*H.capsulatum*	RJ	EPM1025	MK893671	MK893876	Brazil
*A.capsulatus*	RJ	RS01	GU320853	GU321032	Brazil
*A.capsulatus*	RJ	RS09	GU320854	GU321002	Brazil
*A.capsulatus*	RJ	3356	GU320879	GU321033	Brazil
*A.capsulatus*	RJ	TI05	GU320851.1	GU321018.1	Brazil
*A.capsulatus*	RJ	TI01	GU320850.1	GU321017.1	Brazil
*A.capsulatus*	RJ	IGS4_5	GU320845	GU321029	Brazil
*A.capsulatus*	RJ	IT04	GU320846	GU321020	Brazil
*A.capsulatus*	RJ	6503	GU320838	GU321028	Brazil
*A.capsulatus*	RJ	9291	GU320874	GU321005	Brazil
*A.capsulatus*	RJ	3612	GU320881	GU321024	Brazil
*A.capsulatus*	RJ	3688	GU320834	GU321025	Brazil
*A.capsulatus*	RJ	4334	GU320835	GU321026	Brazil
*A.capsulatus*	RJ	4631	GU320836	GU321027	Brazil
*H.capsulatum*	RJ	EPM1018	MK893664	MK893869	Brazil
*A.capsulatus*	RJ	IGS19	GU320855	GU321001	Brazil
*A.capsulatus*	RJ	RPS51	GU320848	GU321030	Brazil
*A.capsulatus*	RJ	RPS86	GU320856	GU321031	Brazil

### ﻿Haplotype networks

**Figure 3.**
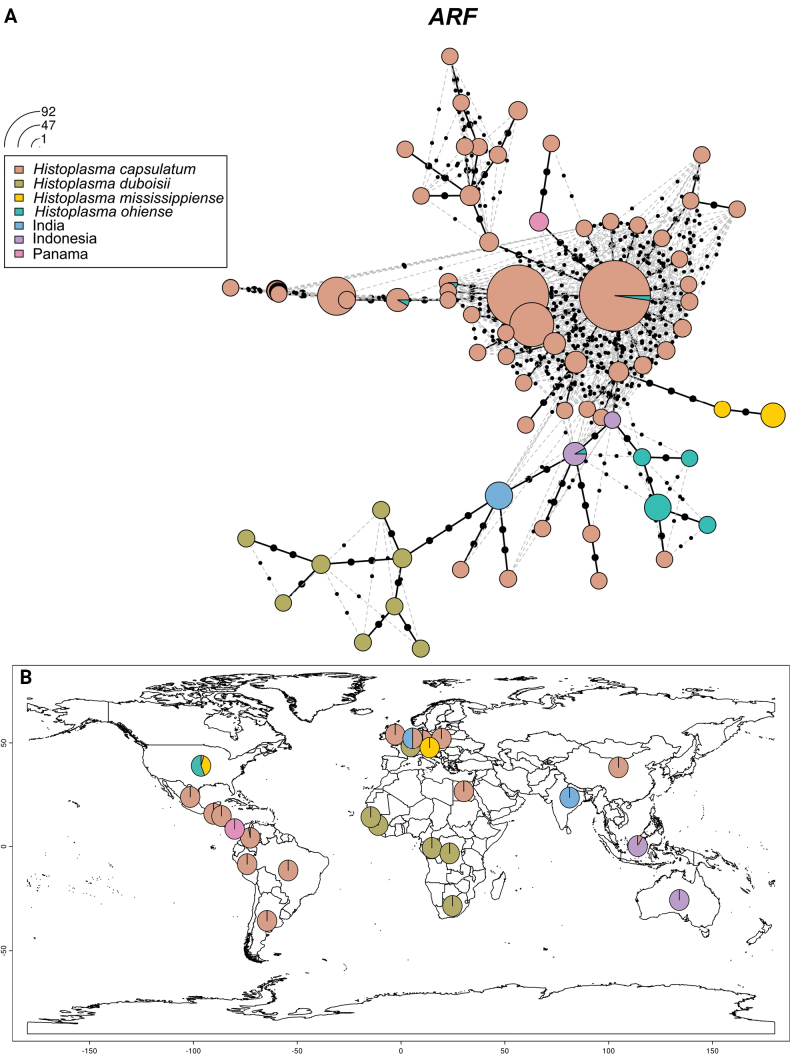
**A** Haplotype networks and distribution patterns of *H.capsulatum* ARF sequences used in this study. The size of the circumference is proportional to the haplotype frequency. Seven colors were coded according to the genetic group representing *Histoplasmaduboisii*, *H.mississippiense*, *H.ohiense*, *H.capsulatum*, India, Indonesia and Panama, respectively. **B** Percentages of individuals per locality assigned to the most probable populations defined by the ARF sequences analysis.

**Figure 4.**
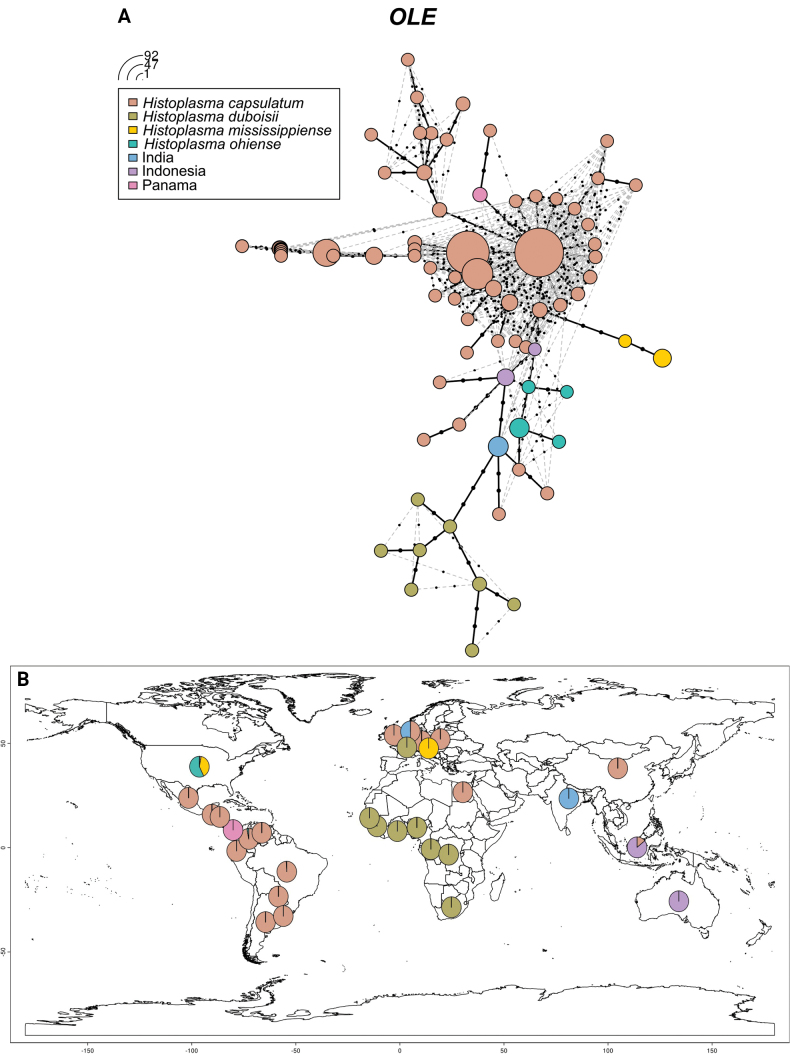
**A** Haplotype networks and distribution patterns of *H.capsulatum* OLE sequences used in this study. The size of the circumference is proportional to the haplotype frequency. Seven colors were coded according to the genetic group representing *Histoplasmaduboisii*, *H.mississippiense*, *H.ohiense*, *H.capsulatum*, India, Indonesia and Panama, respectively. **B** Percentages of individuals per locality assigned to the most probable populations defined by the OLE sequences analysis.

Based on the results above, we classified all the *Histoplasma* strains in this study into six major groups: *H.mississippiense* (NAm 1), *H.ohiense* (NAm 2), *H.duboisii* (Africa), India, Indonesia, *H.capsulatum* / *suramericanum* (LAm A-E, RJ, and other strains). Strains in the group Panama do not form an independent clade in any of the phylogenetic trees. Since Panama contains the type strain of *H.capsulatum* which typifies the genus, understanding its position in the haplotype network is very important. The haplotypes are color coded according to these seven groups. In the haplotype networks of ARF (Fig. 3A) and OLE (Fig. 4A), the strains of *H.capsulatum* / *suramericanum* exhibit the most extensive haplotype diversity, likely due to this group having the largest sample size. Additionally, some strains of this group share haplotypes with certain strains of *H.ohiense* in the ARF haplotype network. In both networks, the *H.ohiense* strains show relatively complex haplotypes. Besides four unique haplotypes clustering together in ARF and OLE, some strains also share haplotypes with *H.capsulatum* and Indonesia only in ARF. In contrast, the *H.mississippiense* group has only two haplotypes. Panama strains have only one unique haplotype in both networks. Similarly, *H.duboisii* strains share the same haplotypes in both networks, of which eight unique haplotypes cluster together. Indian strains have only one haplotype too. In the ARF network, Indonesia strains share haplotypes with *H.ohiense*. In the OLE network, they have two unique haplotypes.

Meanwhile, we mapped the geographic origins of the strains in the seven groups for the ARF (Fig. 3B) and OLE (Fig. 4B) genes separately. The geographic distributions of the strains involved in both genes are almost identical. The strains of *H.capsulatum* are distributed almost globally. The strains *H.mississippiense* have two origins, the United States and Colombia. *H.ohiense* strains also have two origins, the United States and one strain from Austria. Most of the *H.duboisii* strains originate from Africa, although one strain (H88) is recorded from Belgium, which is likely from an immigrant. One strain (H176) diagnosed in the Netherlands forms a sister clade with Indian strains in the ARF tree and clusters within the Indian branch in the OLE tree. Similarly, one strain (H157) from Australia forms a sister clade with Indonesian strains in the ARF tree and clusters within the Indonesian clade in the OLE tree.

### ﻿Geography

**Figure 5.**
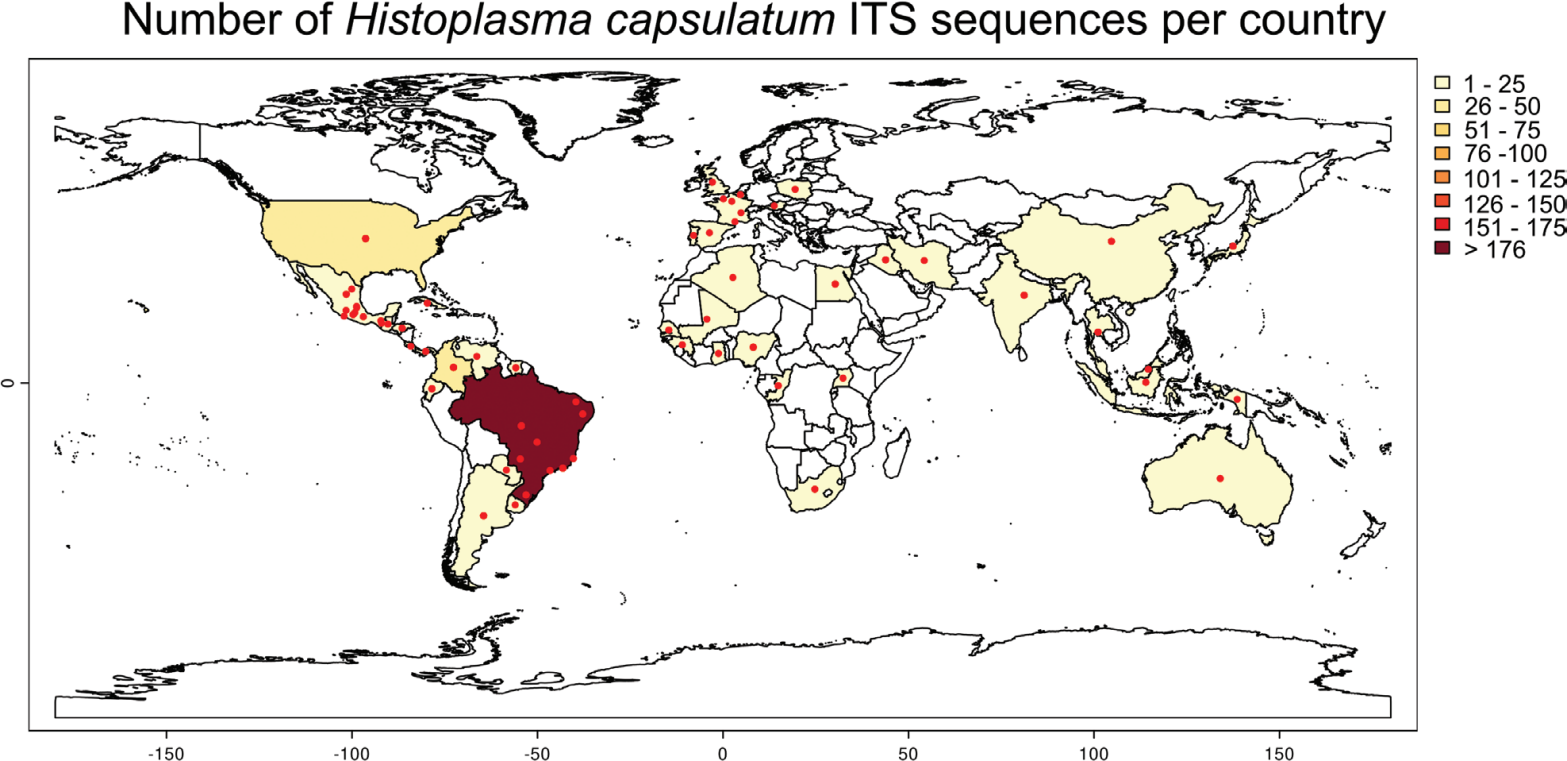
The global distribution of all strains in this study.

This study includes a total of 879 *Histoplasma* strains from 47 countries or regions (Fig. 5), covering all continents except Antarctica. Among them, the strains from South America are the most abundant, with 454 strains, accounting for 51.65% of the total. North America contributes 266 strains, accounting for 30.26% of the total. Africa has 36 strains, representing 4.10% of the total. There are 73 strains from Asia, comprising 8.30% of the total, and 29 strains from Europe, accounting for 3.30% of the total. Only two strains originate from Australia. As of the current study, there have been no reports of *Histoplasma* from Antarctica.

Most strains from Asia lacked protein-coding sequence depositions, so that only ITS rDNA could be included in the analysis. The ITS tree comprised a cluster only containing strains from Asian countries, although lacking significant bootstrap support. The clade referred to as NAm-1 contains, besides strains from the U.S.A., five strains from Iraq. In all trees, the majority of African strains cluster together to form an independent branch (98% BS in ITS, 95% BS in ARF, 97% BS in OLE). Eleven strains from Indonesia formed clades with relatively high support in the ARF (BS 76%, BS 99%, PI 99%), OLE (BS 87%, BS 98%, PI 100%), two genes (BS 99%, BS 99%, PI 100%) and multilocus (BS 100%, BS 100%, PI 100%) trees. No significant group support was achieved with H-antigen and ITS. Sixteen strains from India clustered together almost in all trees with high bootstrap values, including ITS (BS 87%, BS 100%, PI 100%), ARF (BS 95%, BS 99%, PI 100%), OLE (BS 80%, BS 98%, PI 100%), two genes (BS 96%, BS 98%, PI 100%) and multilocus (BS 81%, BS 97%, PI 100%). In all trees, strains from Brazil were scattered over several clades, but the NAm-1, NAm-2, and the African, Indonesian and India clades did not include Brazilian strains.

Given the pronounced clustering with geographical preferences, the North American clades consistently being separate from the Latin American clades, strains from the Central American country Mexico, which borders the U.S.A., consistently clustered with Central and South American strains, defined as the LAm clade. In contrast, three strains from Colombia, 1986, COL_H_001 and COL_H_002, consistently appear in the NAm-2 clade, while remaining Colombian strains are scattered throughout the tree along with strains from South America. The strains of the Panama cluster was located inside the distribution of the L-Am clade.

The majority of African strains clustered together in most of the trees. The ITS tree comprises a total of 30 strains from Africa. Fifteen strains clustered in a single clade with bootstrap support of 98%, along with two strains from the U.S.A. Additionally, 15 strains were scattered in the lower half of the tree and did not form a single clade. In the ARF tree, six strains from Africa clustered in a clade, while four African strains did not form a separate clade. In the OLE tree, twenty one strains from Africa formed an independent clade (BS 97%, BS 99%, PI 100%) without contributions from other origins. This gene is the most effective in distinguishing African strains among all single-gene trees. A similar scenario occurs in the ARF-OLE tree, which contains a total of ten strains from Africa in a clade with high support rate (BS 100%, BS 100%, PI 100%). Six African strains clustered in one clade in multilocus trees with high support rate (BS 99%, BS 98%, PI 100%). The H-antigen sequences failed to effectively resolve the systematic classification of African strains. Although the strains are clustered, the groups lack significant support.

### ﻿Case reports review

As a sample study, we reviewed 50 case reports published from 2023 to 2024 (Suppl. material 1: table S2), involving 50 patients aged between 2 and 83 years, predominantly middle-aged men. Among these patients, 25 had normal immune function, and 25 were immunocompromised. These patients came from various countries, including U.S.A., India, Colombia, Brazil, Venezuela, Bangladesh, Ecuador, Argentina, Nepal, Panama, Cameroon, Nigeria, China, Mexico, Palestine and Australia. Thirty-seven cases were diagnosed as disseminated histoplasmosis, while the remaining cases had infections localized to single organs such as the lungs, trachea, vertebrae, oral cavity, adrenal glands, bones, heart, thyroid, eyes, skin, and tongue. The majority was described with presence of small yeast cells inside macrophages. This indicates that histoplasmosis can have a severe impact on individuals with normal immune function and can affect multiple organs. Based on these 50 case reports, no clinical differences attributable to regional variations in histoplasmosis could be identified. Also for the diseases caused by the North American, Indian (Agrawal et al. 2020) and Indonesian (Baker et al. 2019) genotypes as yet were not reported to differ significantly from classic histoplasmosis. However, strains from tropical Africa are known to cause African histoplasmosis which is characterized by subcutaneous nodules and histopathology comprising large, broad-based budding cells outside macrophages (Develoux et al. 2021). The variety *farciminosum* is known as the cause of histopathological disease in donkeys (Powell et al. 2006), but no sequence was available to draw a solid taxonomic conclusion.

## ﻿Discussion

Primary aim of the present paper is a comparison of trees involving all current barcoding sequence data of *Histoplasma* with earlier, less well-populated molecular data sets, and to retrospectively evaluate their predictive power. However, the current data set is characterized by an enormous overabundance of data from Latin America. This probably does not represent the global diversity of *Histoplasma*, as members of the genus are also known to be prevalent in Africa and Asia (Valero et al. 2018; Lv et al. 2020). An important constraint in collecting *Histoplasma* strains is their classification as Biosafety Level 3 fungi, requiring isolation and cultivation in BSL-3 safety cabinets which are not widely available in all areas where *Histoplasma* is prevalent. Strict transportation conditions have also limited the scientific exchange of *Histoplasma*. The study of (sub) tropical fungal pathogens poses a challenge for many developing countries and contributes to the significant regional underestimation of the prevalence of this fungus.

Another limitation in phylogenetic studies of *Histoplasma* lies in molecular analysis. Although the comparative part of our study encompasses a total of 879 strains, only 274 possess sequences for all four genes required for concordance studies, with 400 strains having sequences for both ARF and OLE. Multilocus sequencing for these fungi is not a standard procedure for most laboratories where *Histoplasma* is endemic. Preservation of strains in publicly accessible collection centers with molecular facilities and safety equipment will enhance future studies.

With these limitations in mind, we selected four genes that are commonly used in phylogenetic studies of *Histoplasma*. Consistent with earlier research (Kiss 2012; Rodrigues et al. 2020), ITS sequences showed poor resolution, producing a limited number of supported clades. rDNA is primarily suitable for identifying *Histoplasma* at the genus level. The use of ITS sequences for phylogenetic analysis of *Histoplasma* is beneficial because ITS sequences represent the most abundantly populated data set for *Histoplasma*, providing a rich resource for research despite its low diversity. The alignment lengths of ARF and OLE data sets were 418 and 407 bp, respectively, and contained more phylogenetic information. The alignment of the diagnostic H-antigen is only 223 base pairs, which is inadequate for phylogeny. Improved primer design to amplify longer fragments is required to enhance the role of H-antigen in *Histoplasma* phylogeny. Alternatively, additional genes such as RPB1, known to significantly affect the phylogeny of other fungi (Kandemir et al. 2022), might be used to explore the diversity of *Histoplasma*.

Only three studies based on genome sequences have been conducted in *Histoplasma* phylogeny, involving a total of 62 genomes (Sepúlveda et al. 2017; Almeida-Silva et al. 2021; Jofre et al. 2022). Given their high information, genome studies will be essential to establish the global diversity of *Histoplasma*.

### ﻿Predictive value of earlier phylogenetic studies

Kasuga et al. (1999) utilized a multilocus sequence typing (MLST, ARF, OLE, H-anti, TUB1) technique to assess the phylogenetic relationships among 46 *Histoplasma* strains from different areas, prior to study representing three varieties: vars *capsulatum*, *duboisii*, and *farciminosum*. The results identified six clades: (i) North American *H.capsulatum* (NAm-1), (ii) North American *H.capsulatum* (NAm-2), (iii) Central American *H.capsulatum* (Panama), (iv) South American *H.capsulatum* group A (SAm-A), (v) South American *H.capsulatum* group B (SAm-B), and (vi) African H.capsulatumvar.duboisii. The concordance among the four gene genealogies suggests that the above six groups have been reproductively isolated from each other for a long time. A few years later, the same authors (Kasuga et al. 2003) introduced additional strains while retaining the original ones, bringing the total number of strains to 137. After the same MLST analysis, the authors redefined eight clades. Three previously recognized clades (NAm-1, NAm-2, Africa) were retained, and clades SAm-1 and SAm-2 were renamed as LAm-A and LAm-B. The previous clade Panama was deleted. Three new clades (Netherlands, Australia, Eurasia) were added. Seven clades should be considered as phylogenetic species, except for the Eurasian clade, as it was considered to have emerged from the largest clade, South American LAm-A. The agent of Egyptian horse disease, classically referred to as H.capsulatumvar.farciminosum, was not recognizable as a monophyletic group, as strains clustered in different clades. Histoplasmacapsulatumvar.duboisii , together with strains previously identified as var. capsulatum, composed a separate African clade. The authors suggested that the three varieties in their classical circumscription are phylogenetically meaningless. Of the seven clades finally distinguished by Kasuga et al. (2003), current data confirmed NAm-1, NAm-2, and Africa-*duboisii*, all showing concordance in three genes. LAm-A and LAm-B had no concordance in all genes and were not separated, while the Netherlands and Australia (possibly concerning immigrants) were represented by single isolates.

Teixeira et al. (2016) further increased the number of analyzed *Histoplasma* sequences to 246 and employed various methods of phylogenetic analysis and population genetics. Their study retained the six clades described by Kasuga et al. (2003), while they suggested that the two Latin American clades comprised at least 6 phylogenetic entities. Among these clades, particularly LAm-A exhibited a complex population genetic structure, supporting at least 4 monophyletic clades named LAm-A1, LAm-A2, RJ, and BAC-1. The LAm-B clade could be divided into two highly supported clades, which were geographically restricted to either Colombia/Argentina or Brazil, respectively. Despite observed structured diversity with a strong geographic component in our current data, clade concordance indicated significant gene flow between South American groups. Multiple mechanisms of genetic exchange can be involved, which is a subject for later studies.

Valero et al. (2018) studied African *Histoplasma* using ITS, RBP1, and OLE genes. In that study, nine strains from Africa clustered in a single clade with a BS support rate of 96%. However, in our ITS tree, these nine strains did not cluster together; only two clustered in the *duboisii* clade (98% BS), while the remaining seven were scattered in the LAm-A clade.

Rodrigues et al. (2020) utilized data from protein-coding loci (ARF, H-antigen, OLE), rDNA barcoding (ITS), AFLP markers, and mating type analysis to assess the genetic diversity, population structure, and identified distinct phylogenetic species among 436 strains of *Histoplasma* from around the world but focused on Brazil. The study distinguished three separate groups in South America, namely LAm-C, LAm-D, and LAm-E, all originating from Brazil. In our data, these groups were difficult to recognize.

In addition to MLST, genomic sequence analysis has been introduced as a next level taxonomic approach in *Histoplasma*. Sepúlveda et al. (2017) performed genome-wide population genetics and phylogenetic analyses with 30 *Histoplasma* isolates representing four endemic areas but with a strong focus on South America. Four phylogenetic species were formally introduced (Sepúlveda et al. 2024), i.e. *Histoplasmacapsulatums. str.* (Panama), *H.mississippiense* (NAm-1), *H.ohiense* (NAm-2), *H.suramericanum* (LAm-A), and one African clade without attribution of a name. In our current data, Lam-A sequences were distributed over several clades. Almeida-Silva et al. (2021) sequenced 18 *Histoplasma* genomes additional to the 30 from earlier research (Sepúlveda et al. 2017) and constructed a genome-level phylogenetic tree. Apart from the four previously recognized clades, this study reinstated LAm-B as an independent clade. In addition, *Histoplasmasuramericanum* was composed of at least two populations, one in the northern part of South America, and another in the southern portion of the continent. Jofre et al. (2022) sequenced genomes of 16 *Histoplasma* isolated from India and combined those with the 30 genomes of Sepúlveda et al. (2017) to construct a genomic level phylogenetic tree. A new phylogenetic species was recognized in India. This conclusion matches with our barcode and genealogical concordance study, where a clearly separate Asian (India / Indonesia) species was distinguishable.

In summary, populations of *Histoplasma* show strong regional structuring, which has led authors to distinguish several entities with decreased gene flow. In main traits, all authors agree that *Histoplasma* comprises several species, while in contrast *H.farciminosum* is indistinguishable from *H.capsulatum* / *suramericanum*. The expansion of the number of studied strains reveals that the sample has less common genetic events, indicating that not all genetically different groups deserve recognition as species. *Histoplasmacapsulatum*/*suramericanum* is a species that occurs globally, while the regional species *H.ohiense*, *H.mississippiense* (Tenório et al. 2024) and *H.duboisii* (Ameni et al. 2022) are consistently deviating. A separate Asian species (Jofre et al. 2022) might deserve formal recognition.

### ﻿Ecology and clinical significance

*Histoplasma* is an environmental pathogen (Carpouron et al. 2022) that grows on guano of bats, pigeons, and chickens in sheltered habitats. It is designated to infect the local animals, develop an invasive yeast phase inside macrophages, and return to the environment via feces and carcasses (Taylor et al. 2022). Human infections are fulminant in patients with impaired acquired immunity (Damasceno et al. 2019), but disseminated histoplasmosis may also occur in immunocompetent individuals. It has been suggested that the severity of infection partly depends on the size of the inoculum inhaled (Ling et al. 2018). Humans are not the preferred host of the fungus. The sheltered habitats of the fungus explain the fragmented, regionally differing population structure. For many of these populations, clinical differences are being investigated (Damasceno et al. 2019; Jones et al. 2020), statistically significant deviations between groups as yet being unconvincing, with the exception of the variety *duboisii*. *Histoplasmacapsulatum* is a pulmonary, intracellular disease finally leading to cutaneous pustules, while infections caused by var. duboisii are distinguished by a high propensity for involvement of skin, lymph nodes, and bone. Osseous lesions are typically osteolytic processes that can simulate cancer (Pakasa et al. 2018; Amona et al. 2021; Develoux et al. 2021). Phylogenetically, it is a distinct lineage (Ameni et al. 2022) at short distance from others (Almeida-Silva et al. 2021). The disseminated form of the disease can manifest as skin lesions, which have been confirmed through direct examination in HIV-infected patients (Wahyuningsih et al. 2021). The histological features of infection caused by *Histoplasmaduboisii*, which are also distinctive from those of *H.capsulatum*, are characterized by large ovoid to globose yeast-like cells measuring 8–15 μm in diameter with broad-based budding. By comparison, there are currently no data supporting a clinical or histological distinction of disease caused by the *H.ohiense*, or *H.mississippiense*, which thus could be viewed as cryptic species. Whether or not the Asian genotype is clinically similar to *H.capsulatum* (Sayal et al. 2003; Krıshnamurthy et al. 2021) needs to be established. The current database (Suppl. material 1: table S2) for distinguishing the clinical, immunological, and ecological characteristics among patients infected with different *Histoplasma* species may lack sufficient statistical power to distinguish among the three species. A multicenter, prospective study which identifies key epidemiological and clinical variable with isolates collected for whole genomic sequencing, proteomics, mating studies, and host-fungus *in vitro* properties may be able to further distinguish clinical, physiological, epidemiological, and immunological properties among these different species.

### ﻿Taxonomy

This study primarily focuses on reevaluating previously defined phylogenetic species based on gene sequences. For whole genome phylogeny, three studies have as yet been conducted in *Histoplasma*, involving a total of 62 genomes. Sepúlveda et al. (2017) distinguished NAm-1, NAm-2, LAm and the African clade as the main entities, similar to the main conclusions in our gene studies. This suggests a broad correspondence of genome analysis and barcoding studies in *Histoplasma*.

The primary and most significant limitation in the phylogenetic understanding of *Histoplasma* diversity lies in sample collection. Although the present comparison encompasses a total of 879 strains, only 274 possess sequences for all four genes, with 400 strains having sequences for both ARF and OLE. The absence of sequences for the majority of strains is primarily due to their unavailability in public databases. Currently, only 39 living strains are stored in the CBS fungal collection. Preserving strains in publicly accessible collections will facilitate future retrospective studies. Another important constraint in collecting *Histoplasma* strains is their classification as Biosafety Level 3 organisms, requiring isolation and cultivation in Biosafety Level 3 cabinets. This poses a challenge for many developing countries and contributes to the significant underestimation of *Histoplasma* prevalence in Asia and Africa.

Through analysis of multiple gene trees, we found that the Africa, NAm-1, NAm-2, India and Indonesian clades are supported in most gene trees in addition to the main species, i.e. the global species containing the ex-type of *H.capsulatum*. Most clusters composing the different variants of LAm showed profuse genetic recombination and are therefore judged to represent a single species. For example, sequences in the clusters of LAm-A and RJ are distributed over several clusters throughout the entire tree with the four genes applied in this study. Clusters LAm-D and LAm-E do not form supported clades in all trees. The strains of LAm-B cluster into two well-supported branches in both the ARF and two gene trees, supporting the suggestion of Teixeira et al. (2016) to divide the LAm-B clade into two subclades. Additionally, one of the genome studies (Almeida-Silva et al. 2021) also suggests retaining the phylogenetic species status of LAm B. However, the genealogical concordance analysis indicates that only a subset of LAm-B strains exhibit consistent relationships in ARF and OLE phylogenies. Therefore, we postpone a final decision on LAm-B until after analyzing a larger number of strains with better global representation, and the influence of the bias of South American material is evaluated. As for LAm-C, the strains cluster together in all phylogenetic trees, but without obtaining effective support. Hence, future studies should introduce genome data for LAm C to further determine its classification status.

## ﻿Conclusion

The instability of medically relevant fungi can be illustrated by comparison of the first and 4^th^ editions of the Atlas of Clinical Fungi (de Hoog et al. 1995, 2020): during these 25 years, 81.5% of all names underwent some kind of change, being synonymized, reallocated, or split up in molecular siblings. This certainly has many reasons as explained above. However, the present study underlines that early molecular phylogenies of *Histoplasma* remained relatively stable in consecutive studies while applying more data, although further subdivisions of main groups might gain limited support. The question of which recognized clades should be regarded as intraspecific lineages or rather as individual species remains a matter of debate. The only area where our conclusions deviated from that of some of the earlier papers concerned the possible distinction of the three isolates in the Panama cluster and *H.suramericanum*. The latter species appears to be global rather than limited to the South American continent, and it is questionable whether the small Panama cluster is sufficiently different to deserve separate species status. This question has large practical consequences, because the Panama / adjacent countries cluster contains the epitype strain of *H.capsulatum*. We think the necessity to change name of the global population to *H.suramericanum*, and to restrict *H.capsulatum* to a very small cluster in Panama, is not convincingly proven.

## ﻿Nomenclature

The oldest description of the fungus causing histoplasmosis we have located is by Rivolta (1873), who described it from equine lymphangitis in Egypt but did not provide a formal binomial scientific name, but referred to it variously as “Criptacoccus del pus farcino” (p. 583), “Criptococchi nel farcino” (p. 524), and “Criptococchi nel pus farcino” (p. 575, in the legend to the plates). The species was first given the formal species name *Cryptococcusfarciminosus* by Rivolta & Micelloni (1883, as “criptococcus farciminosus”). No physical original material has been traced, but while no figure was included in the 1883 paper, the 1873 publication was referred to and therefore counts as original material for the purpose of typification and so must be designated as **lectotype**, which we do here. Rivolta’s fungus was subsequently recognized as belonging to *Histoplasma* by Ciferri and Redaelli (1934) and combined into that genus as *Histoplasmafarciminosum*.

Weeks et al. (1985) aimed at designating dried material with a metabolically inactive lyophilized culture from a horse in Egypt as neotype of the epithet *farciminosum*: CBS 536.84 (= ATCC 58332 = H90 = CDC B-3786), but this act was invalid as a neotype is not to be designated while original material is extant (Art. 9.8). In order to minimize possible confusion, here we formally designate this same material as an interpretive type (i.e. an **epitype**) for the illustration here designated as lectotype for *Cryptococcusfarciminosus*.

*Cryptococcusfarciminosum* has been recombined into nine additional genera (de Hoog et al. 2020). CBS 536.84, although unstably classified in different phylogenetic trees, shares identical sequences with many isolates in ITS and OLE phylogenetic trees. In the ARF and the two-gene OLE-ARF tree, it clusters with several strains from Europe. This name antedates *H.capsulatum* by 32 years, but as this would be an unwelcome name change for one of the most familiar fungal pathogens, a formal proposal to protect the name *H.capsulatum* over *H.farciminosum* and any other competing names that may be discovered in the future is being made under Art. F.2.1.

Darling (1906) described *H.capsulatum* from a patient in Panama who had arrived there from Martinique three months earlier. As it is unclear whether any slides or cultures have been preserved, the only definite original material is the figure in Darling’s publication, which we therefore designate as a **lectotype** of the name here. Kasuga et al. (2003) mentioned three strains from Panama, of which H81 (= ATCC 26028) is frequently taken as a reference strain for *H.capsulatum* (Sepúlveda et al. 2017). Berliner (1968) reported that H81 is G184B from human sputum sent by a M.H. Shacklette (Washington) under no. C121A but its provenance is unclear. In order to preclude further confusion, we decided to designate CBS 145499 from Guatemala as an **epitype** of the lectotype illustration for Darling’s name here.

Additionally, CBS 136.72 (= ATCC 22635) is frequently mentioned as an ex-type strain of *H.capsulatum*, e.g. in MycoBank and in the GenBank Taxonomy Browser. It concerns an isotype mating partner of the sexual morph described as *Ajellomycescapsulatus* (Kwon-Chung et al. 1974). However, sequencing established that this represents the North American *H.ohiense* rather than *H.capsulatum* as typified here. The placing of *Ajellomycescapsulatus* as a synonym of *H.ohiense* does not have any nomenclatural repercussions as that epithet is pre-occupied in *Histoplasma*.

Given the fact that *Histoplasmasuramericanum* (LAm) is doubtfully separated from *H.capsulatum*, that name is regarded as synonymous.

Moore (1934) described *Posadasiapyriformis* as a species close to Darling’s *H.capsulatum* from a strain originally reported from Iowa in the U.S.A. by Hansmann and Schenken (1934). A more extended description was published one year later (Moore 1935), clearly underlining a close affinity of the two species; *P.pyriformis* was recombined as *Histoplasmapyriforme* by Dodge (1935). Given the North American origin of the strain, identity with one of the *H.capsulatum* molecular siblings is likely, but none of Moore’s original material other than perhaps an illustration in Hansmann & Schenken’s paper we have not seen, is known to be preserved. Consequently, the identity of Moore’s species cannot be established.

The variety *duboisii* is confirmed in the present and earlier phylogenetic studies (e.g., Almeida-Silva et al. 2021). The disease caused by this species deviates from the prevalent type of histoplasmosis as seen on the American continent (Ocansey et al. 2022). Also, the tissue phase is different due to the production of large budding cells. The phylogenetic, clinical and microscopic differences support the maintenance of this entity at the species level.

In summary, the following species are recognized here and listed with their synonyms. Names in the complex whose applications remain unclear are also added for completeness.

### ﻿*Histoplasmacapsulatum* Darling – J. Amer. Med. Assoc. 46: 1285, 1906, nom. prot. prop.

≡ *Moniliacapsulata* (Darling) Lindner & Knuth – Z. Infektionskrankh. 17: 299, 1916 [n.v.].

= *Cryptococcusfarciminosus* Rivolta & Micelloni – Giorn. Anat. Fisol. Patol. Anim. Dom. 15: 162, 1883; as “*criptococcus farciminosus*”. Type: Rivolta, Dei Paras. Veg.: fig. 153b, 1873 (**lectotype designated here**, MBT 10022527); Egypt: isolated from horse with epizootic lymphaginitis, 1983, comm. S.A. Selin, CDC B-3786 [dried culture]. **Epitype designated here**, CDC B-3786, MBT 10022528). Ex-epitype cultures ATCC 58332, CBS 536.84.

≡ *Cryptococcusrivoltae* Farmi & Arnch – Centralbl. Bakteriol. Parasitenk. 1 Abt. 17: 597, 1895 (name change).

≡ *Saccharomycesfarciminosus* (Rivolta & Micelloni) Tokishige – Zentralbl. Bakteriol. Parasitenk., Abt. 1, 19: 112, 1895.

≡ *Leishmaniafarciminosa* (Rivolta & Micelloni) Galli-Valerio – Centralbl. Bakteriol. Parasitenk., Abt. 1, 44: 577–582, 1909.

≡ *Endomycesfarciminosus* (Rivolta & Micelloni) Nègre & Bouquet – Bull. Soc. Pathol. Exot. 10: 274, 1917.

≡ *Parendomycesfarciminosus* (Rivolta & Micelloni) Mello & L.G. Fern. – Arq. Hig. Pat. Exot. 6: 29, 1918.

≡ *Grubyellafarciminosa* (Rivolta & Micelloni) M. Ota & Langeron – Ann. Parasitol. Humaine Comp. 3: 78, 1925.

≡ *Coccidioidesfarciminosus* (Rivolta & Micelloni) Vuill. – Champ. Paras. Myc. Homme Anim. p. 140, 1931.

≡ *Torulopsisfarciminosus* (Rivolta & Micelloni) F.P. Almeida – Ann. Fac. Med., São Paulo 9: 76, 1933.

≡ *Histoplasmafarciminosum* (Rivolta & Micelloni) Redaelli & Cif. – Boll. Sierot. Milan. 10: 851, 1934; as “*farcinimosus*”.

≡ *Zymonemafarciminosum* (Rivolta & Micelloni) C.W. Dodge – Med. Mycol.: 169, 1935.

≡ Histoplasmacapsulatumvar.farciminosum (Rivolta & Micelloni) Weeks et al. – Mycologia 77: 969, 1985; nom. inval. (Art. 41.5).

= *Histoplasmasuramericanum* Sepúlveda et al. – mBio 8(6): e01339-17, 13, 2017; nom. inval. (Art. 40.7).

≡ *Histoplasmasuramericanum* Sepúlveda et al. – mSphere 9(6): e00009-24, 11, 2024. Type: CBS 145499, strain 3/11, Guatemala.

**Type.** Darling, J. Amer. Med. Assoc. 46: 1284, fig. 1, 1906, **lectotype designated here**, MBT 10022526; Panama: isolated from human with histoplasmosis CBS 145499, metabolically inactive culture preserved in liquid nitrogen, **epitype designated here**, MBT 10021411. Ex-epitype culture: CBS 145499.

### ﻿*Histoplasmamississippiense* Sepúlveda et al. – mSphere 9(6): e00009-24, 9, 2024.

≡ *Histoplasmamississippiense* Sepúlveda et al. – mBio 8(6): e01339-17: 12–13, 2017 nom. inval. (Art. 40.7). Type: CBS 145498, strain CI#19, Missouri, USA.

### ﻿*Histoplasmaohiense* Sepúlveda et al. – mSphere 9(6): e00009-24, 11, 2024.

≡ *Histoplasmaohiense* Sepúlveda et al. – mBio 8(6): e01339-17: 13, 2017, nom. inval. (Art. 40.7). Type: CBS 145496, strain Cl#17, Missouri, USA.

= *Emmonsiellacapsulata* Kwon-Chung – Science, N.Y. 177: 368, 1972. Type: USA: BPI 71811, Arkansas, Miller County, isolated from soil samples under bird roosts, K.J. Kwon-Chung (ATCC 22635, ATCC 22636, CBS 136.72, CBS 137.72 – ex-type cultures of opposite mating types).

≡ *Ajellomycescapsulatus* (Kwon-Chung) McGinnis & Katz – Mycotaxon 8: 158, 1979.

### ﻿*Histoplasmaduboisii* Vanbreus. – Ann. Soc. Belge Méd. Trop. 32: 578, 1952.

≡ Histoplasmacapsulatumvar.duboisii (Vanbreus.) Ciferri – J. Amer. Med. Assoc. 2: 342, 1960.

**Type.** CBS 215.53, isolated from guinea pig previously injected with a strain from human with African histoplasmosis, Congo, R. Vanbreuseghem (RV 4754).

### ﻿Names of uncertain application

*Saccharomycesequi* Marcone – Atti Reale Ist. Incoragg. Napoli 8–6: 1–19,1895.

*Cryptococcustokishigei* Vuillemin ex Guéguen – Champ. Paras. l’Homme Anim. Domest.: 108, 1907 ≡ *Parendomycestokishigei* (Vuillemin ex Guéguen) Mello –Arq. Hig. Pat. Exot. 6: 295, 1918.

*Posadasiapyriformis* M. Moore – Ann. Missouri Bot. Gard. 21: 347, 1934 ≡ *Histoplasmapyriformis* (M. Moore) C.W. Dodge – Med. Mycol.: 155, 1935.

## ﻿Additional information

### Adherence to national and international regulations

All the fungal strains used in this study have been legally obtained, respecting the Convention on Biological Diversity (Rio Convention).

### Author ORCIDs

Yu Quan https://orcid.org/0000-0002-3103-3118

Xin Zhou https://orcid.org/0000-0003-2094-7288

Ricardo Belmonte-Lopes https://orcid.org/0000-0002-3122-0271

Na Li https://orcid.org/0000-0002-4713-0705

Retno Wahyuningsih https://orcid.org/0000-0002-3294-5792

Anuradha Chowdhary https://orcid.org/0000-0002-2028-7462

David L. Hawksworth https://orcid.org/0000-0002-9909-0776

Sean Zhang https://orcid.org/0000-0003-1166-2563

Marcus de Melo Teixeira https://orcid.org/0000-0003-1763-3464

Daniel Matute https://orcid.org/0000-0002-7597-602X

Sybren de Hoog https://orcid.org/0000-0002-5344-257X

Dong Wu https://orcid.org/0000-0002-2842-1144

### Data availability

All of the data that support the findings of this study are available in the main text or Supplementary Information.

## Supplementary materials

Supplementary material 1 Supplementary figures and tables Data type zip Explanation note **fig. S1.** Phylogenetic tree of *Histoplasma* based on Internal Transcribed Spacer (ITS) sequence, obtained by maximum likelihood. **fig. S2.** Phylogenetic tree of *Histoplasma* based on ADP-ribosylation factor (ARF) sequence, obtained by maximum likelihood. **fig. S3.** Phylogenetic tree of *Histoplasma* based on delta-9 fatty acid desaturase (OLE) sequence, obtained by maximum likelihood. **fig. S4.** Phylogenetic tree of *Histoplasma* based on H-antigen precursor (H-anti) sequence, obtained by maximum likelihood. **fig. S5.** Phylogenetic tree of *Histoplasma* based on two gene sequences (ARF and OLE), obtained by maximum likelihood. **fig. S6.** Phylogenetic tree of *Histoplasma* based on multilocus sequences (ITS, ARF, OLE and H-anti), obtained by maximum likelihood. **fig. S7.** Phylogenetic tree evolved genealogical concordance analysis based on ARF sequences, obtained by maximum likelihood. **fig. S8.** Phylogenetic tree evolved genealogical concordance analysis based on OLE sequences, obtained by maximum likelihood. **table S1.** Strains of *Histoplasma* analysed in this study. **table S2.** Fifty case reports of histoplasmosis between 2023 and 2024. https://binary.pensoft.net/file/1336809This dataset is made available under the Open Database License (http://opendatacommons.org/licenses/odbl/1.0/). The Open Database License (ODbL) is a license agreement intended to allow users to freely share, modify, and use this Dataset while maintaining this same freedom for others, provided that the original source and author(s) are credited.Yu Quan, Xin Zhou, Ricardo Belmonte-Lopes, Na Li, Retno Wahyuningsih, Anuradha Chowdhary, David L. Hawksworth, J. Benjamin Stielow, Thomas J. Walsh, Sean Zhang, Marcus de Melo Teixeira, Daniel Matute, Sybren de Hoog, Dong Wu
