# Supplementary figures and images for: ﻿Potential predictive value of phylogenetic novelties in clinical fungi, illustrated by Histoplasma
Source: IMA Fungus. 2025 May 23;16:e145658. doi: 10.3897/imafungus.16.145658 (PMC12125600; doi:10.3897/imafungus.16.145658)

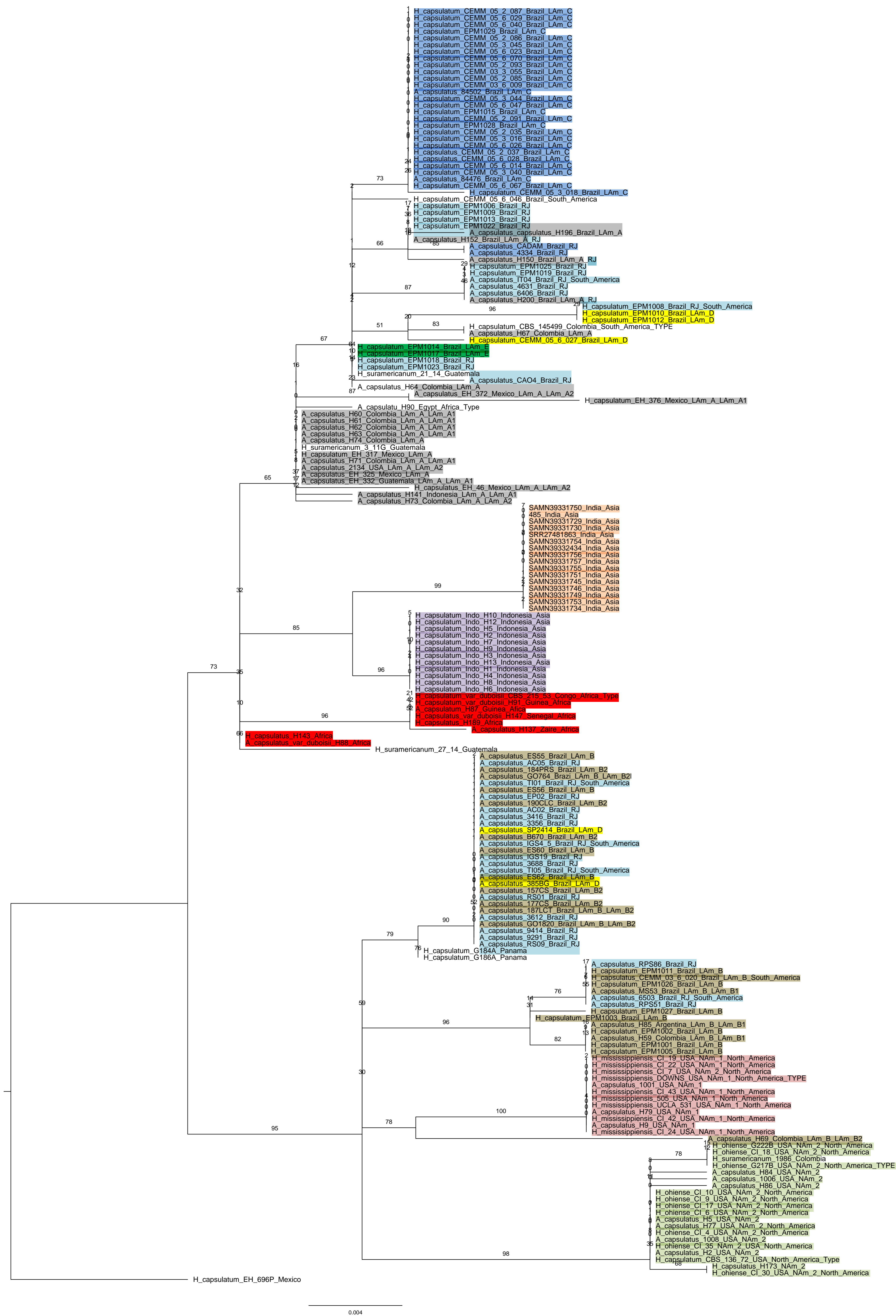

Supplement: Supplementary material 1 — Supplementary figures and tables [file imafungus-16-e145658-s001.zip › supprement/S7. ARF 2.pdf]

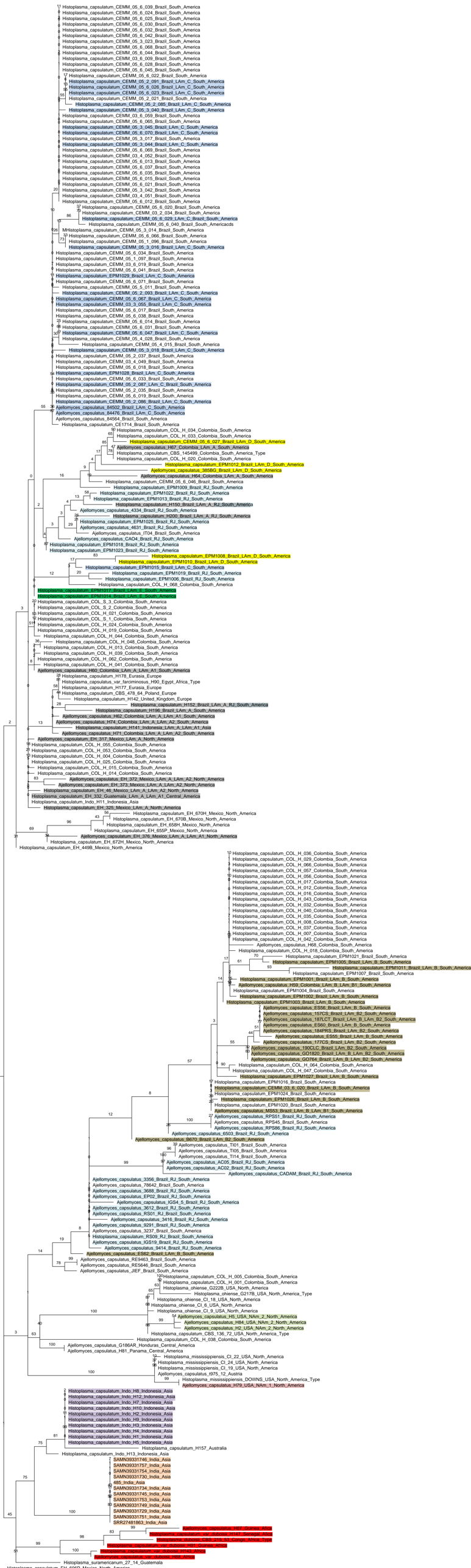

Supplement: Supplementary material 1 — Supplementary figures and tables [file imafungus-16-e145658-s001.zip › supprement/S6 combine.result.pdf]

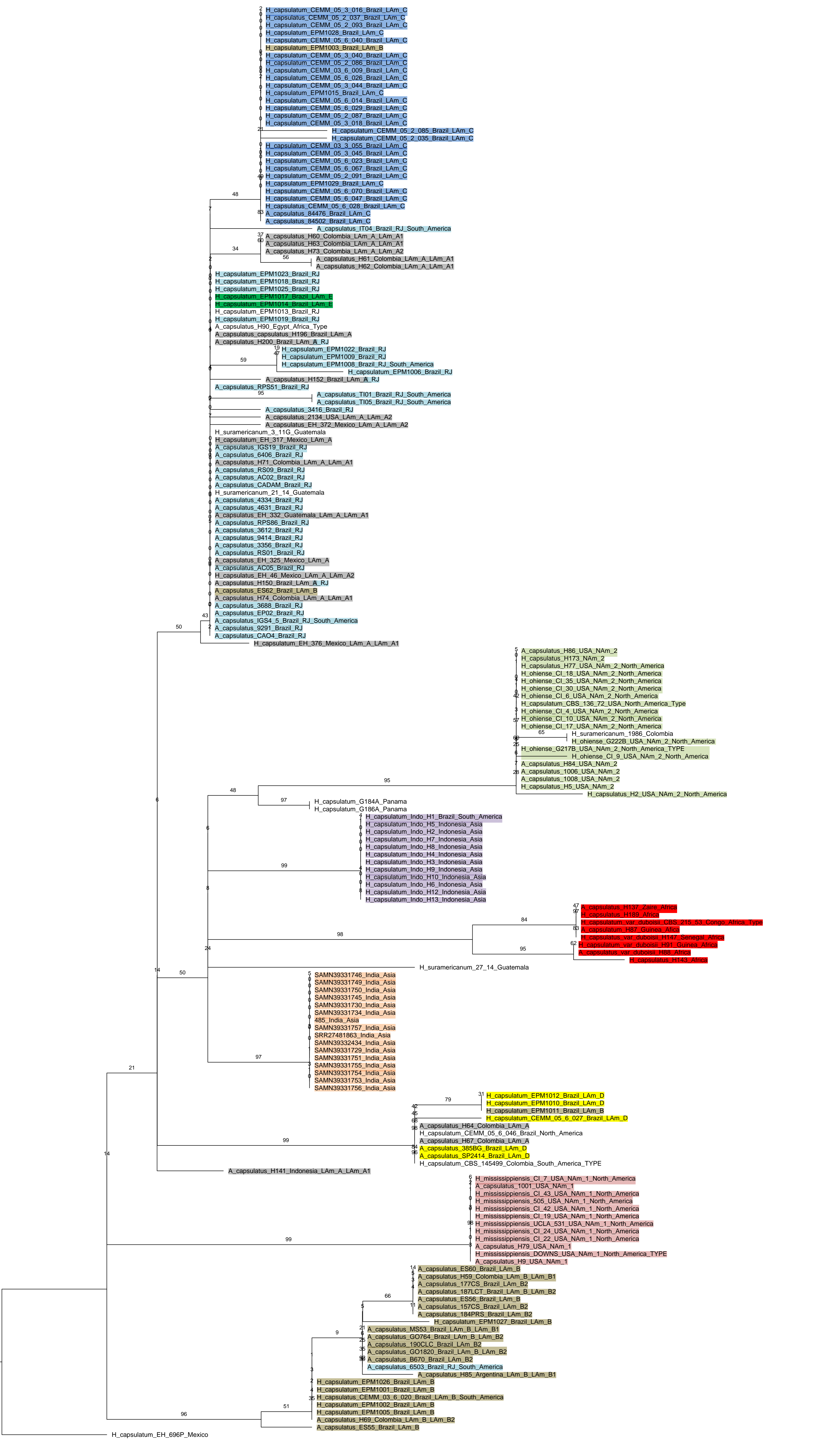

Supplement: Supplementary material 1 — Supplementary figures and tables [file imafungus-16-e145658-s001.zip › supprement/S8 ole 2.result.pdf]
