# Supplementary material for: ﻿Potential predictive value of phylogenetic novelties in clinical fungi, illustrated by Histoplasma
Source: IMA Fungus. 2025 May 23;16:e145658. doi: 10.3897/imafungus.16.145658 (PMC12125600; doi:10.3897/imafungus.16.145658)

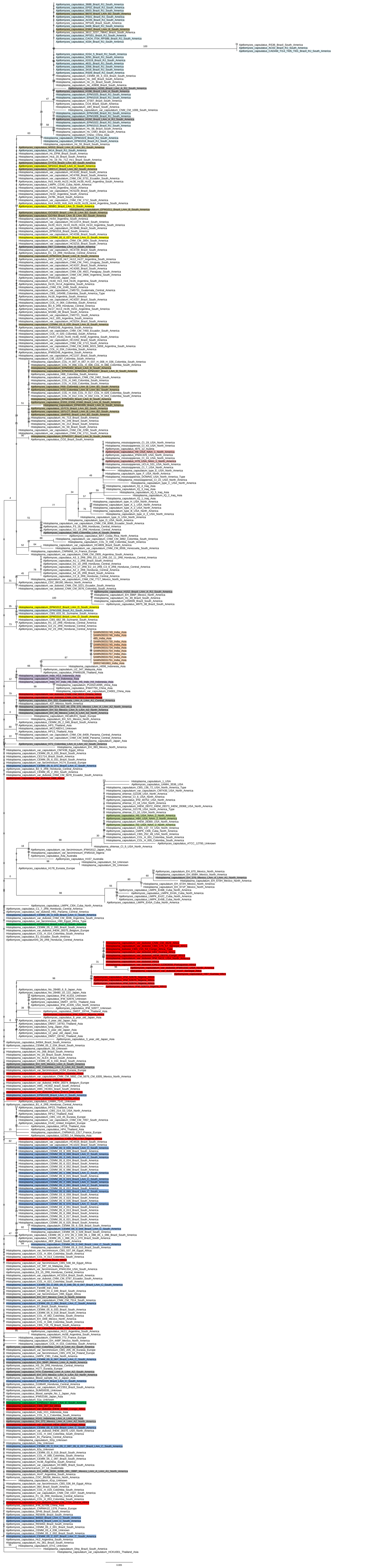

Supplement: Supplementary material 1 — Supplementary figures and tables [file S1 ITS.result.pdf › supprement/S1 ITS.result.pdf]
